# Not All Sequence Tags Are Created Equal: Designing and Validating Sequence Identification Tags Robust to Indels

**DOI:** 10.1371/journal.pone.0042543

**Published:** 2012-08-10

**Authors:** Brant C. Faircloth, Travis C. Glenn

**Affiliations:** 1 Department of Ecology and Evolutionary Biology, University of California Los Angeles, Los Angeles, California, United States of America; 2 Department of Environmental Health Science, University of Georgia, Athens, Georgia, United States of America; Michigan State University, United States of America

## Abstract

Ligating adapters with unique synthetic oligonucleotide sequences (sequence tags) onto individual DNA samples before massively parallel sequencing is a popular and efficient way to obtain sequence data from many individual samples. Tag sequences should be numerous and sufficiently different to ensure sequencing, replication, and oligonucleotide synthesis errors do not cause tags to be unrecoverable or confused. However, many design approaches only protect against substitution errors during sequencing and extant tag sets contain too few tag sequences. We developed an open-source software package to validate sequence tags for conformance to two distance metrics and design sequence tags robust to indel and substitution errors. We use this software package to evaluate several commercial and non-commercial sequence tag sets, design several large sets (max_count_ = 7,198) of edit metric sequence tags having different lengths and degrees of error correction, and integrate a subset of these edit metric tags to polymerase chain reaction (PCR) primers and sequencing adapters. We validate a subset of these edit metric tagged PCR primers and sequencing adapters by sequencing on several platforms and subsequent comparison to commercially available alternatives. We find that several commonly used sets of sequence tags or design methodologies used to produce sequence tags do not meet the minimum expectations of their underlying distance metric, and we find that PCR primers and sequencing adapters incorporating edit metric sequence tags designed by our software package perform as well as their commercial counterparts. We suggest that researchers evaluate sequence tags prior to use or evaluate tags that they have been using. The sequence tag sets we design improve on extant sets because they are large, valid across the set, and robust to the suite of substitution, insertion, and deletion errors affecting massively parallel sequencing workflows on all currently used platforms.

## Introduction

Synthetic, oligonucleotide sequence identification tags (sequence tags) can be attached to individual pieces of DNA allowing pooling and sample tracking during massively parallel sequencing (MPS) [1]–[3]. Sequence tags enable efficient distribution of the output from these platforms among many individually identifiable samples rather than extensive, deep sequencing of single individuals or mixed samples. Thus, the ability to tag and track sequenced DNA from many individuals in multiplex increases the efficiency of MPS when the genomes being sequenced are small [4] or when researchers want to apportion the output of MPS platforms among smaller genomic regions of many individuals [5]–[7].

Groundbreaking prior work introduced the idea of sequence tagging by incorporating tags to sequence reads using polymerase chain reaction (PCR) primers and DNA ligation [1]–[3]. Yet, early sequence tags were designed for specific platforms and platform-specific error patterns, and few tag sets were created to address the complement of errors (insertions, deletions, and substitutions) affecting the uniqueness of each tag sequence across the suite of current sequencing platforms. Errors can also be introduced to sequence tags during tag synthesis and strand replication (library preparation or template amplification), in addition to DNA sequencing.

Errors in sequence tag synthesis occur during the coupling reaction, when DNA bases are being joined to form the desired oligonucleotide strand [8]. Coupling errors produce n-1, n-2, and n-3 congeners containing deletion errors throughout the oligo [9], [10]. Relatively expensive purification techniques remove most of these congeners, particularly the n-2 and n-3 varieties, but some n-1 congeners remain, even with increasingly sophisticated purification methods (e.g., HPLC) [11]. Thus, all synthetic oligonucleotides have the potential to contain deletion errors, and this potential increases significantly when expensive purification is not used. However, expensive purification techniques are increasingly cost prohibitive as the number of required sequence tags or adapters containing tags increases, and HPLC purification can introduce additional problems if sequence tagged adapters or sequence tagged primers are sequentially purified [12] without accounting for carryover.

Errors in strand replication often occur during the amplicon generation or library preparation process (*c.f.* [13]), because researchers use thermostable DNA polymerases and PCR to generate amplicons, increase library concentration by ligation-mediated PCR, or add sequence tags to adapter-ligated fragments. Thermostable DNA polymerases predominately incorporate substitution errors to DNA strands during replication [14], [15], although most DNA polymerases can produce new DNA strands containing insertion or deletion errors at a lower frequency [15], [16]. The error rate is template- and polymerase-dependent, and modern proof-reading DNA polymerases having exonuclease activity exhibit low rates of nucleotide incorporation error, suggesting that these types of enzymes should be used in all amplicon sequencing and library preparation procedures [17]. Similar synthesis errors accrue during downstream template amplification (i.e., emulsion PCR [emPCR] for 454, Ion Torrent and SOLiD platforms or cluster formation for Illumina), but this is generally less of a problem because sequences are determined from the consensus of many molecules on one particle or in one cluster.

Sequencing errors occur on all MPS platforms, but the type of errors and the error rates vary across MPS platforms [18]–[25]. Sequencing errors on platforms from Roche 454, Applied Biosystems (Ion Torrent), and Pacific Biosciences largely consist of insertion and deletion errors, whereas sequencing errors on platforms from Illumina and Applied Biosystems (SOLiD) are generally substitutions [26], [27]. Single-read sequencing error rates vary from 0.5–5% [20], [21], [25], [28] on Roche, Illumina, and Applied Biosystems platforms to 18% on the Pacific Biosciences platform [23]. Sequencing error rates are not uniformly distributed across sequence reads from platforms that amplify the templates (e.g., Illumina, Ion Torrent and Roche) with most errors occurring at the beginning and end of reads [18], [22], [29]. This biased distribution of sequencing errors along a read affects sequence tags immediately adjacent to or far from the start of the sequence read [30] to a greater degree than sequence tags offset from 5′ or 3′ ends.

Synthesis, replication, and sequencing errors negatively impact the utility of sequence tags because they change the basepair composition of individual tags by inserting bases to, substituting bases within, or deleting bases from the identifying sequence. All three types of error can cause one tag to appear identical to another (crossover) or sufficiently alter a sequence tag such that it is unrecognizable (loss) and untraceable to the source material. A uniformly distributed error rate of 1.0% during an MPS sequencing run producing 10^6^ reads, each having an 8 bp sequence tag, results in approximately 77,000 reads (8%) having more than one error within the sequence tag (Figure S1). Probability ensures that longer sequence tags, which allow multiplexing of more samples, are affected by sequencing error to a greater degree, and tags of longer length should have greater minimum distance from all tags in the set.

**Figure 1:**
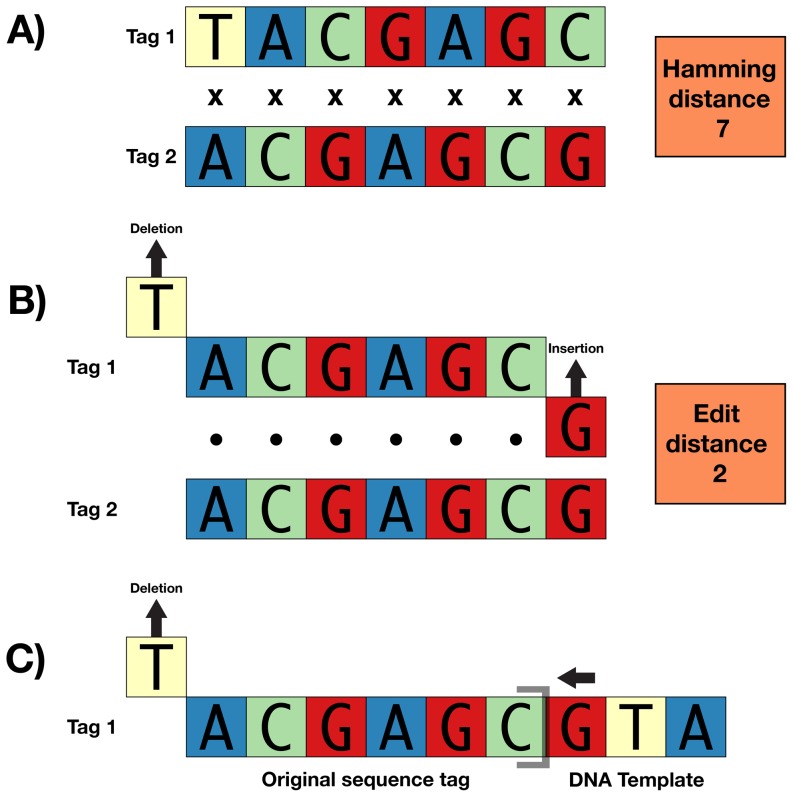
Insertion and deletion errors violate the codeword scheme and reduce the utility of Hamming-based tags. Panel (A) shows two sequence tags that are different from one another by seven substitutions (Hamming distance = 7) – a distance more than sufficient to differentiate tags in the presence of substitution errors. However, these same two tags have an edit distance of two (B) – meaning that a total of two insertions, substitutions, or deletions can turn Tag 1 into Tag 2 and confuse samples. Although it seems improbable that two indels or substitutions would occur in a sequence tag, consider the third case (C) in which a single deletion event at the 5′ end of a sequence tag adjoining DNA template beginning with 5′ guanine confuses Tag 1 with Tag 2. Edit metric sequence tags of distance three or greater would mitigate this mistake.

**Figure 2:**
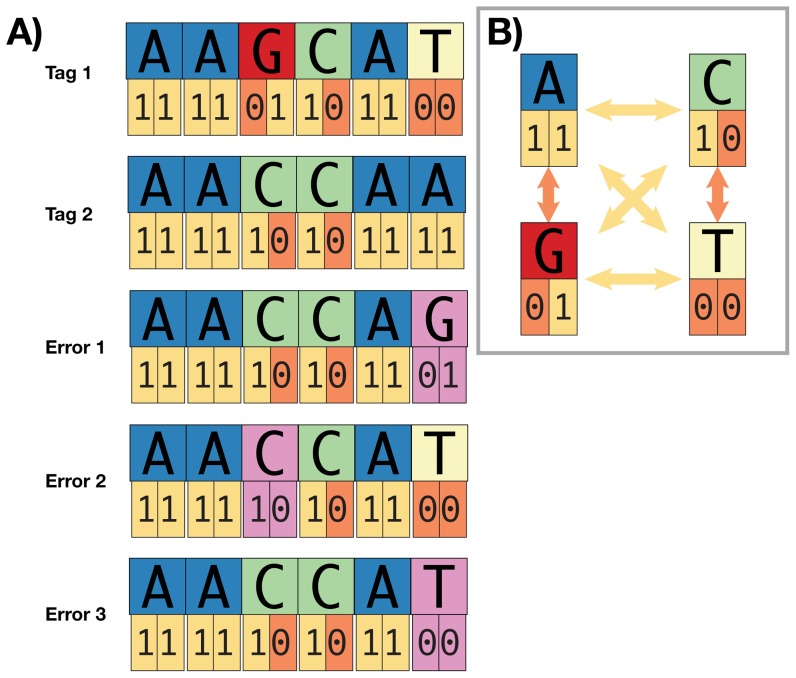
Using Hamming codes to design binary encoded sequence tags when synthesis, replication, or sequencing errors mutate the nucleotide sequence reduces the number of single-base errors that are correctable during downstream demultiplexing. Here, we show two sequence tags (Tag 1 and Tag 2) and both their nucleotide and binary encodings. Tag 1 and Tag 2 have a Hamming distance of four between their binary representations and a Hamming distance of two between their nucleotide representations. Error 1 is correctable to Tag 2, because a single nucleotide substitution (in purple) results in a single, binary difference (11 versus 01) between Error 1 and Tag 2, and single binary errors are correctable when tags are at least three binary differences from each other. Error 2 and Error 3 tags also exhibit a single nucleotide substitution (in purple) but two binary differences from Tag 1 and two binary differences from Tag 2. Because there is more than a single binary difference, we cannot determine whether the source tag was originally Tag 1 or Tag 2, we cannot correct the error, and we must discard the read. More generally, because of the binary encoding and the Hamming distance between tags (Hamming distance four between binary representations, Hamming distance two between nucleotide representations), we can correct single binary errors seen in the substitutions around the perimeter of inset (B), but we cannot correct double binary errors across the diagonals of inset (B). Because these single nucleotide, double binary substitutions (i.e., across the diagonals) comprise two of six potential substitution mutations, we cannot correct 33% (2/6) of single nucleotide substitution errors.

Using error-correction schemes, researchers can construct sequence tags that are more robust to synthesis, replication, and sequencing errors (i.e., minimizing crossover and loss) while also allowing the correction of certain types of errors. Hamady et al. [31] used Hamming codes [32] to develop a set of error-correcting sequence tags with which they successfully tracked a large number of reads in multiplex (see also [33]). However, Hamming codes assume that the errors occurring within each sequence tag are only substitutions [34], [35]. Insertion and deletion errors violate the codeword scheme and reduce the utility of Hamming-based tags when commercial synthesis does not completely remove n-1 congeners, standard *Taq* polymerase is used during strand replication, or sequence data are generated on platforms incorporating insertion and deletion errors (Figure 1; [36]). Additionally, when Hamming-distance tags are constructed using a binary representation of each base (e.g., T = 00; G = 01; C = 10; A = 11), which we define as “binary encoding” (Figure S2), 33% of substitution errors, while detectable, are uncorrectable because sequencing errors occur among actual nucleotides (Figure 2; [37]). Thus, sequence tags appropriately designed using Hamming codes should use nucleotide representations of each base rather than their binary encoding [37].

Sequence tags based on the edit metric or Levenshtein distance [38], [39] are superior to Hamming-distance tags, because edit metric sequence tags are robust to the types of errors introduced by oligonucleotide synthesis, replication, and DNA sequencing: insertions, deletions, and substitutions. Edit metric sequence tags allow for error correction according to the following formulas [38]–[40]:
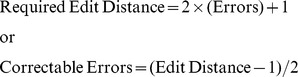
Thus, we can correct up to two sequencing errors in sequence tags from a set having an edit distance of five. Although edit metric sequence tags are provided by several commercial (e.g., Roche 454, Inc.) and non-commercial sources [40], [41], there are few available methods (*c.f.* [29]) of generating sets of edit metric-based sequence tags. Furthermore, current methods may generate tags that do not correctly follow the edit metric (Table 1), and current methods are best suited to generating sequence tag sets comprising tags of shorter length (≤8 nt). The continually increasing output of MPS platforms suggests that large collections of edit metric sequence tags will be essential to distributing output across smaller genomes, select genomic regions, and populations of individuals.

**Table 1:** Commercial and non-commercial sequence tag sets and the conformance of each to the stated or assumed distance metric (edit or Hamming).

Class	Set Name	Length (nt)	N_tags_	Design Algorithm	Minimum Distance		Pair Violations	Tags≥D_expected_	Comments
					exp	obs			
Contain Violations	Illumina TruSeq sRNA	6	48	Hamming	3	2	2	47	Some tags violate expected Hamming distance
	Hamady et al. 2007^1^	8	1544	Hamming	3	4/2^2^	-	1544	Only corrects 66% of errors
	Meyer et al. 2010^3^	6	75	Edit	3	2	40	49	Some tags violate expected edit distance
	Meyer et al. 2010^3^	8	711	Edit	3	2	551	429	Some tags violate expected edit distance
	Adey et al. 2010^4^	9	96	Edit	4	2	58	64	Some tags violate expected edit distance
Correct Hamming distance	Illumina TruSeq RNA and DNA	6	27	Hamming	3	3	-	27	
	Meyer et al. 2008^5^	7	52	Hamming	3	3	-	52	
	Meyer et al. 2008^5^	8	130	Hamming	3	3	-	130	
Correct edit distance	Qiu et al. 2003	6	21	Edit	3	3	-	21	
	Frank 2009^6^	6	81	Other	2	2	-	81	Design algorithm similar to edit distance 2
	Illumina Nextera DNA^7^	8	8/12	Edit	3	3	-	8 or 12	
	Frank 2009^2^	8	760	Other	2	2	-	760	Design algorithm similar to edit distance 2
	Roche 454 MID Extended	10	151	Edit	4	4	-	151	
	Roche 454 RL-MID Extended	10	132	Edit	4	4	-	132	
Designed for this publication	EDDITTAG	6	61	Edit	3	3	-	61	
	EDDITTAG	7	211	Edit	3	3	-	211	
	EDDITTAG	8	531	Edit	3	3	-	531	
	EDDITTAG	9	1,936	Edit	3	3	-	1,936	
	EDDITTAG	10	7,198	Edit	3	3	-	7,198	

1 Hamady *et al.* [31] tags are from the nmeth.1184-S1.pdf supplementary file.

2 Hamady *et al.* [31] tags are Hamming distance 4 from one another in binary encoding but Hamming distance 2 from one another in nucleotide encoding.

3 We generated Meyer *et al.* [29] tags using: ‘python create_index_sequences.py -l <length> -d 3’.

4 Adey *et al.* [41] tags are from the gb-2010-11-12-r119-s3.pdf supplementary file.

5 Meyer *et al.* [3] tags are from the nprot.2007.520-S1.doc supplementary file.

6 We generated Frank [42] tags using: ‘barcrawl -l <length> -m 3’. BARCRAWL uses a hybrid approach to create distance between tags while accounting for a single deletion.

This is similar to an expected edit distance of two.

7 Illumina Nextera tags are incorporated to either end of the template strand in combinatorial fashion to identify up to 96 samples.

Here, we introduce *EDITTAG*, a collection of tools for testing sequence tags for conformance to the edit or Hamming distance metric, generating edit metric sequence tags, and programmatically applying sequence tags to PCR primers and platform-specific sequencing adapters. *EDITTAG* differs from similar programs by providing: (1) a method to check the conformity of previously designed tags, adapters, linkers, or primers to the edit metric; (2) a method to generate edit metric sequence tags of arbitrary length; (3) methods for prepending sequence tags to amplification primers and inserting tags into platform-specific sequencing adapters; and (4) multiprocessing support to speed tag generation when tag lengths are long (≥8 nt).

We use components of EDITTAG to validate a number of existing sequence tag sets provided by commercial and non-commercial sources, design several sets of edit metric sequence tags of varying edit distance, and integrate a subset of edit metric sequence tags to Epicentre Nextera adapters, Illumina TruSeq adapters, and PCR primers. We then validate this subset of tags by sequencing across the indices of indexed adapters and sequence-tagged PCR primers on the Illumina (GAIIx and HiSeq 2000) and Roche 454 (FLX Titanium) platforms.

## Materials and Methods

EDITTAG provides a suite of Python (http://www.python.org) programs for: validating sequence tags for conformance to the edit or Hamming distance metrics, designing edit metric sequence tags, and incorporating sequence tags to amplicons or platform-specific sequencing adapters. We describe implementation details for each of these EDITTAG processes, and we follow each description with the steps we followed to implement or validate each process.

### Sequence Tag Validation

The validate_edit_metric_tags.py program within EDITTAG checks existing tag sets, alone or incorporated into PCR primers or sequencing adapters, for conformance to the edit metric by performing pairwise, edit distance comparisons between each tag in the input set and all other tags in the set. In short, the program iterates through the set of tags input; computes the pairwise edit distance between all tags in the set using either a C-based Python module or a pure-Python method; and outputs either the minimum distance of the set, those tag pairs having an edit distance less than the minimum expected, or the edit distance between all members of a set, depending on the output options selected by the user. This program is also capable of computing the Hamming distance between sequence tag inputs based on selection of the Hamming algorithm in place of the edit distance algorithm by the user.

We used validate_edit_metric_tags.py to test the conformance of eight existing sequence tag sets available from commercial (Illumina, Inc. and Roche 454, Inc.) and non-commercial sources [29], [31], [40]–[42] to their respective distance metric (Hamming or edit) by appropriately formatting an input file for these tags (File S1) and inputting this file to the program. We used the tag-rescanning feature of design_edit_metric_tags.py (described below) to determine the number of tags in these sequence tags sets having minimum edit distances of three and five.

### Sequence Tag Design

Technically, designing error-correcting sequence tags is a matter of generating all *n*-length combinations of [A,C,G,T]; filtering tags based on subjective or platform-specific criteria including removal of: combinations containing homopolymer runs, combinations with undesirable base composition, or individual tags that are perfect self-complements; and iteratively comparing each tag in the remaining group against all other tags in the remaining group to create the largest set that maintains some minimum edit distance. Practically, the process is more complex because the design of sequence tag sets requires comparison of all tags in the candidate set to all other tags in the candidate set. Given sequence tags of sufficient length, this requirement rapidly approaches the limits of desktop computation. For example, the full set of 10 nucleotide tags contains 1,048,576 members, which requires 550 billion pairwise edit distance comparisons across all tags in the candidate set. If storage of each result requires 8 bits, then storing the entire array requires approximately 500 GB - a daunting object with which to work. Additionally, this considers only the first stage of processing and ignores the additional computational and storage overhead required to select and test subsets of edit metric sequence tags.

Thus, we modified the approach used by the lexicode algorithm [43] to speed up processing, reduce memory consumption, and enable parallelization of jobs across multiple processors. Briefly, our approach first generates all *n*-length combinations of [A,C,G,T]. Then, if the remaining group is sufficiently large, we apportion tags into discrete batches of 25,000 tags, and we distribute each batch among the available number of processing cores to (optionally) remove those tags having problematic composition (homopolymers, improper GC, perfect self-complements). After filtering, we rebuild the set of candidate tags returned from each processing core, and we create the following data structure, where the 0th position of each “row” below is a sequence tag “key” to which we pair a “value” comprising a list of all tags in the set:
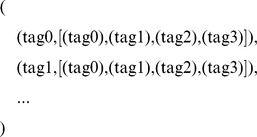
If this data structure is sufficiently long (more than 500 “rows” as illustrated above), we apportion the structure into batches containing 500 “rows”, and we distribute each batch among the available number of processors. Iterating over each row, we then compute the edit distance between the “key” and all sequence tags in the value list using either a C-based Python module (http://pylevenshtein.googlecode.com) or a pure-Python method. To reduce memory consumption when iterating over millions of tags, we produce a summary vector for each key giving the count of all other sequence tags having values that fall within edit distance categories (0, 1, 2, …, *N*), and we use the 0-indexed position of the count in the vector to denote the edit distance. Thus, the vector:
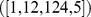
corresponds to a key having a single tag edit distance 0 from the key, 12 tags edit distance one from the key, 124 tags edit distance two from the key, and five tags edit distance three from the key. We then reduce the data by keeping only those keys having the maximum count of comparisons at the minimum desired edit distance, a technique that allows us to reduce the remaining number of pairwise comparisons over the entire data set by approximately 99% (estimated from the generation of eight nucleotide, edit distance three tags).

After reducing the data, for each key we compute the edit distance between the key and all sequence tags in the value; we drop any tags in the value less than the desired edit distance; and we iterate over the remaining tags in the value, retaining only those tags that are also the desired edit distance from one another. Finally, we determine the count of remaining tags in the value list for each key, and we return the key (and its values) having the largest value list. Additionally, we include an option that quickly returns subsets of keys within this final set having edit distances from the key at values greater than the minimum desired edit distance.

We used this approach to design sets of edit metric sequence tags ranging from four to 10 nucleotides in length and having edit distances of three. We used the shortcut method described above to select subsets, within each of these sets, having edit distances from four to nine. After creating these edit distance tags, we validated each set of resulting tags for conformance to the edit metric using validate_edit_metric_tags.py, the program described in the previous subsection.

### Sequence Tag Application

EDITTAG provides two convenience programs for integrating sequence tags to platform-specific adapters and PCR primers. The first program (add_tags_to_primers.py) is meant primarily for integration of sequence tags to PCR amplicons when designing sequence-tagged PCR primers. In brief, this program adds sequence tags to the 5′ ends of both upper and lower PCR primers, optionally removes common bases between each sequence tag and primer sequence, optionally prepends both primers with a sequence (GTTT) promoting +A addition [44] to facilitate adapter ligation, uses Primer3 [45] to evaluate tagged primers for complementarity problems and the presence of hairpins, and outputs all tagged primers to an sqlite (http://www.sqlite.org) database or comma-separated file for subsequent evaluation and selection.

The second program (add_tags_to_adapters.py) simply integrates designed sequence tags to adapters and/or primers by inputting the list of desired sequence tags, the adapter/primer sequence 5′ of the sequence tag location, and the adapter/primer sequence 3′ of the sequence tag location. This program is largely meant to reduce mistakes when manually positioning sequence tags within large numbers of adapters or primers.

### Testing Sequence Tag Integration to PCR Primers

To test the design and resulting utility of PCR primers sequence-tagged using the helper program, we integrated the entire set (n = 164) of 10 nucleotide, edit distance five sequence tags (File S2) to primers amplifying the *rbcLa* locus in land plants [46], [47]. We used the resulting database to select 95 hairpin-free, sequence tagged primers (File S3) which we had commercially synthesized, adding a single 3′ phosophorothioate linkage to each oligo (Integrated DNA Technologies, Inc.). We used these primers to amplify the *rbcLa* locus in 190 tropical forest tree species (2×95 reactions) in a reaction mixture containing 5.0 µL CTAB-extracted [48], purified (AMPure) DNA, 0.3 µM KAPA dNTP mix, 0.2 µM each primer, 1× KAPA HiFi PCR Buffer, 0.5 U KAPA HiFi HotStart polymerase and the following touchdown PCR thermal profile: 95°C for 30 s; 20 cycles of 95°C for 30 s, 66°C for 30 s minus 0.25°C per cycle, 72°C for 1.5 min; 20 cycles of 95°C for 30 s, 60°C for 30 s, 72°C for 1.5 m; 72°C for 15 min. Following PCR, we visualized amplicons by running 7 µL of PCR product on 1.5% agarose gels for 90 minutes at 100 V and staining with ethidium bromide.

We cleaned PCR amplicons and normalized amplicon concentrations across samples using SequalPrep normalization plates (Invitrogen, Inc.), combined sequence-tagged PCR amplicons from a 96-well plate into a single pool, and concentrated the pool using a SpeedVac. Prior to sequencing, we used T/A ligation to add standard 454 GS FLX Titanium sequencing adapters to the 5′ and 3′ ends of each amplicon pool [49]. We quantified the resulting adapter-ligated amplicon pools using qPCR (KAPA Biosystems), we combined amplicon pools at equimolar ratios, and we sequenced amplicon pools using a portion of one 1/8^th^ plate of a 454 GS FLX Titanium sequencing run (UCLA Genotyping Core). We demultiplexed the resulting sequence data using demuxipy (https://github.com/faircloth-lab/demuxipy/); combined the read counts by sequence tag from each pool of *rbcLa* amplicons to minimize the variance introduced to counts by differences in template quality, template quantity, and PCR; averaged the count of reads per sequence tag across pools; and computed fold difference between the average number of reads per sequence-tag and the global average number of reads.

### Validating Edit Metric Tag Addition to Nextera-style Adapters

To validate edit metric sequence tags incorporated to Nextera-style sequencing adapters, we first removed the Epicentre-provided IDX1 and IDX8 adapters from the Nextera barcoding kit to ensure the edit distance of the remaining set (*n* = 10 adapters) was three. We then created 14 new Nextera adapter sequences by incorporating six nucleotide, edit metric three sequence tags to each adapter, and we used validate_edit_metric_tags.py to ensure we maintained an overall edit distance of three among all members of the set (File S4). We commercially synthesized and HPLC-purified these new adapters (Integrated DNA Technologies, Inc.), and we incorporated each indexed adapter to target enriched [50] genomic DNA using PCR, according to the Nextera manual (Epicentre Biotechnologies). Following PCR, we quantified the indexed libraries using qPCR (KAPA Biosystems), pooled sequencing libraries at equimolar concentrations into groups of 12, and sequenced the pooled libraries using two lanes of an Illumina GAIIx DNA sequencer (LSU Genomics Facility). Because we were interested in validating our ability to sequence across these indices and because we wanted to fairly compare our ability to sequence across edit metric and “standard” (Hamming distance) sequence tags, we demultiplexed sequence data using the standard Illumina pipeline, counted, and compared the number of reads assigned to each sequence tag.

### Validating Edit Metric Tag Addition to TruSeq-style Adapters

To validate edit metric sequence tags integrated to TruSeq-style sequencing adapters, we used the helper program (add_tags_to_adapters.py) to incorporate 10 nt sequence tags of edit distance five to a set of 135 TruSeq-style adapters (File S5). We commercially synthesized all 135 adapters (Integrated DNA Technologies, Inc.), with a replicate subset of 24 that were HPLC-purified using a randomization protocol to ensure adapters did not follow each other on the HPLC (eliminating relevant carry-over), and we conducted two experiments.

In the first experiment, we focused on a subset of adapters where the first 6 nt of the 10 nt tag conforms to a minimum edit distance of 3 (BFIDT-000 to BFIDT-045). We made an equimolar pool of the 24 adapters, and we used this adapter pool to construct a library with a single genomic DNA sample using Illumina TruSeq reagents (leaving out the standard Illumina adapters). We then pooled this mixed library with a subset of Nextera-style adapters (total library mass = 1% TruSeq style; 99% Nextera-style), and we sequenced libraries using a single lane of a GAIIx (see details above). We demultiplexed sequence data using the standard Illumina pipeline, counted, and compared the number of reads assigned to each sequence tag.

In the second experiment we incorporated 12 EDITTAG indexed adapters and 12 Illumina TruSeq indexed adapters to DNA libraries using a modified version of an on-bead library preparation method [51] and reagents from New England Biolabs. Following preparation, we quantified libraries using qPCR (Kapa Biosciences, Inc.), normalized library concentration across samples, and enriched individual or pooled libraries for ultra-conserved elements using 2560 probes [50], [52]. Following PCR recovery and Qubit quantification of the target-enriched libraries, we pooled libraries at equimolar ratios, assuming an average fragment size of 350 bp, and we sequenced replicate library pools using two lanes of an Illumina HiSeq 2000 DNA sequencer (Cofactor Genomics, Inc.). Because we were interested in validating our ability to sequence across these indices and because we wanted to fairly compare edit metric and Hamming distance sequence tags we demultiplexed sequence data using a modification of the standard Illumina pipeline and compared the number of reads assigned to EDITTAG-designed and Ilumina TruSeq sequence tags. We also included, in one sequencing lane (L007), several (*n* = 52) additional libraries identified by sequence-tagged adapters designed using EDITTAG (File S5) at equimolar ratios to other libraries in the pool, and we compared the total number of reads across all libraries having EDITTAG-designed sequence tags (*n* = 64) to all libraries having Illumina TruSeq sequence tags (*n* = 12).

## Results

We validated several (*n* = 14) sets of pre-existing sequence tags to ensure that all pairwise comparisons within these tag sets were greater than the minimum expected edit or Hamming distance (Table 1, Table S1, File S6). Several freely available sets of edit metric sequence tags [41] or edit metric sets output by tag design programs [29] contained pairwise comparisons below the minimum expected edit distance (Figures S3, S4). Only those tags provided by Qiu et al. [40] and Roche, Inc. maintained a minimum edit distance sufficient to correct one error (edit distance ≥3) across all pairwise comparisons (Figures S5, S6, S7). Sequence tags designed by BARCRAWL were equal to or greater than a minimum edit distance of two (Figure S8), a result predicted by their design scheme. Readers should note that BARCRAWL does not explicitly use the edit metric as its design algorithm nor does BARTAB attempt error correction during demultiplexing. As a result, sequence tags designed using BARCRAWL are robust to insertion, deletion, or substitution errors, but they should not be used with correction algorithms that assume the edit metric, unless the tag set is culled to remove those tag pairs with edit distance ≤3.

Hamming-distance sequence tags from Meyer et al. [3] conformed to their expected minimum Hamming distance. Although the binary encoded Hamming-distance sequence tags from Hamady et al. [31] conform to their expected minimum Hamming distance, the binary encoding of each tag allows only 66% of errors to be corrected (Figure 2). Several commercial sequence tags provided in the TruSeq sRNA library preparation kit (Illumina, Inc.), the sequences of which researchers may integrate to adapters for use with DNA or cDNA libraries, do not conform to the expected, minimum, pairwise Hamming distance, potentially violating the codeword scheme when IDX41 is combined with either IDX11 or IDX31 (Figure S9).

**Figure 3:**
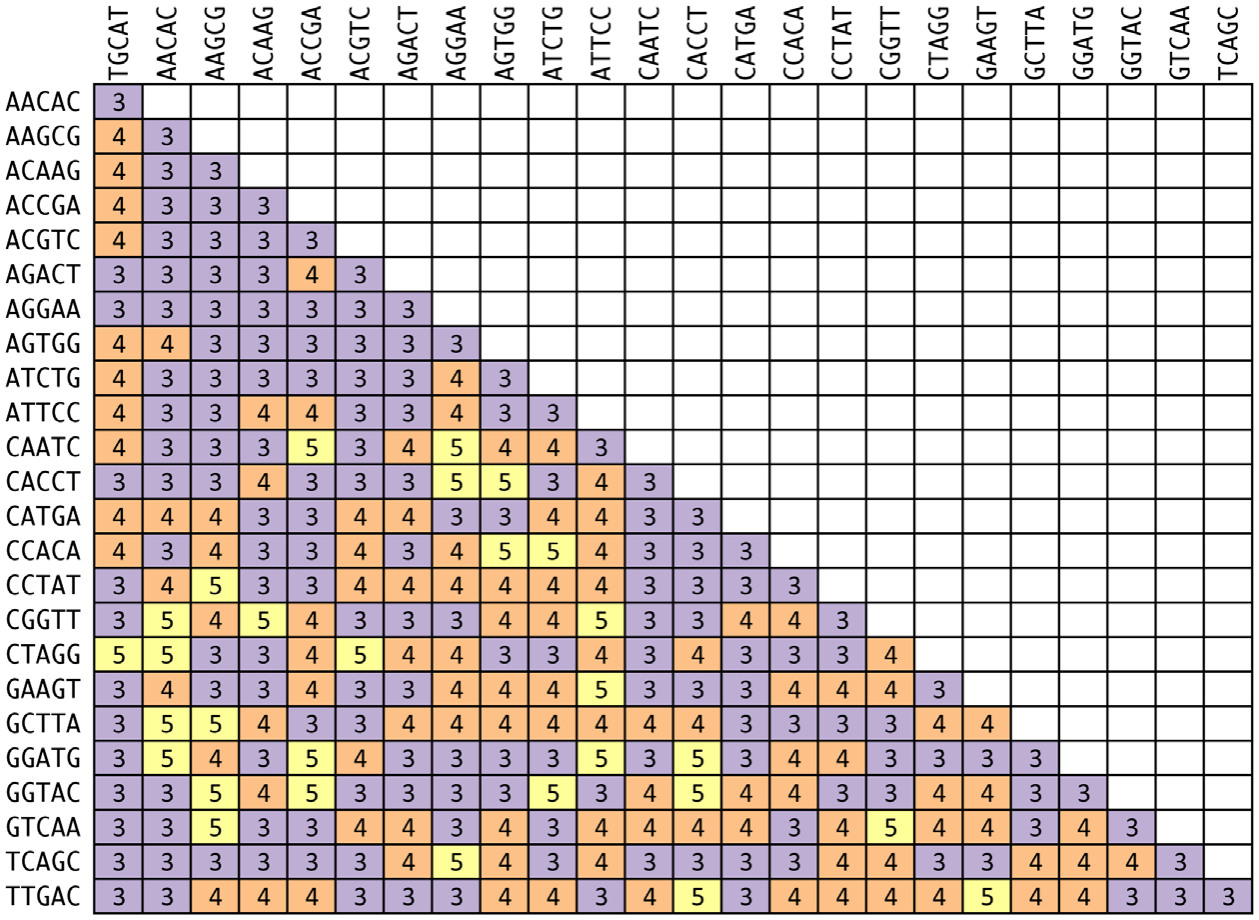
Pairwise edit distance between 25 tags of five nucleotides in length and edit distance three designed using EDITTAG.

We designed several large sets (Table 1, Table 2) of sequence tags of four to 10 nucleotides in length and having a minimum edit distance of three, and we selected all subsets of these sequence tags having edit distances at values greater than the minimum distance within each length category (File S7). We tested the conformance of EDITTAG-designed sequence tags to the edit metric by analyzing all resulting tag sets using our method to compute the minimum, pairwise edit distance between members of a given tag set (validate_edit_metric_tags.py). All tag sets contained members having observed edit distances equal to or greater than the minimum expected edit distance (e.g., Figure 3, Table 1).

**Table 2:** Counts of four to 10 nucleotide, ≥3 edit distance sequence tags sets designed using EDITTAG.

Code Sizes		Edit Distance						
		3	4	5	6	7	8	9
**ID Tag Length**	**4**	7	-	-	-	-	-	-
	**5**	25	7	-	-	-	-	-
	**6**	61	15	5	-	-	-	-
	**7**	211	41	11	4	-	-	-
	**8**	531	103	24	8	3	-	-
	**9**	1936	301	62	18	6	3	-
	**10**	7198	971	164	40	14	5	3

We did not include, in any set, sequence tags having >2 homopolymers, GC content outside the range 40%<GC<60%, or perfect self-complementarity.

We successfully amplified the *rbcLa* locus using each of the 95 primers integrating 10 nt, edit distance five sequence tags (Figure S10). After sequencing, we recovered data from all samples amplified using sequence-tagged primers, and the average fold-difference of read counts per sequence tagged primer did not differ from one (Figure S11), suggesting that incorporation of edit metric sequence tags to primers did not affect amplification or sequencing.

**Figure 4:**
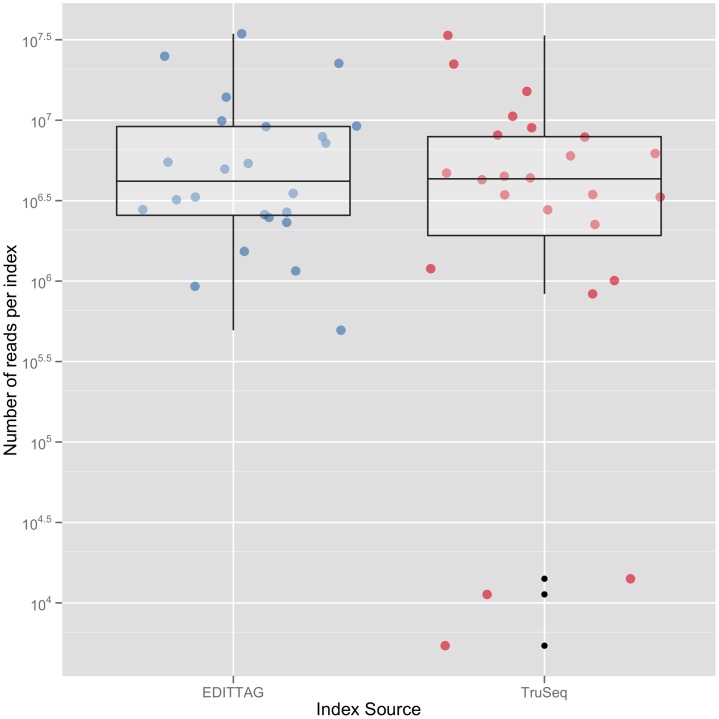
Number of HiSeq reads returned for libraries prepared using Illumina TruSeq adapters versus libraries prepared using adapters integrating edit metric sequence tags designed using EDITTAG.

We successfully sequenced and assigned samples to bins for the 10 nt, edit metric tags incorporated into custom adapters designed for the Nextera (v1; Epicentre Inc.) library preparation system (File S4) and 10 nt edit metric tags incorporated into TruSeq-style adapters with both 6 nt and 10 nt index reads (File S5). The number of reads we recovered from indexed Nextera samples did not differ between the Epicentre indices and the extended set of EDITTAG indices (Figure S12). The number of reads assigned to the 24 tags of the pooled adapter set varied significantly (Figure S13a), but when we ligated individual tags (rather than ligating an equimolar pool of tags) to template molecules during library preparations and directly compared sequence tags design using EDITTAG to Illumina TruSeq indexes, we did not detect a difference in performance (Figure 4, Figure S13b). Additional sequence tags designed using EDITTAG exhibit performance equivalent to commercially supplied indices (Figure S14).

## Discussion

Researchers should validate the codeword scheme of sequence tags incorporated into adapters or PCR primers. Our validation of existing sequence tag sets and/or design methods suggests some sources of sequence tags contain errors (Table 1), and that judicious removal of individual tags violating a particular codeword scheme can yield valid, albeit smaller, tag sets (Table 1, Table S1). Commercial sources of sequence tags are not free of these errors. The effects of set corruption on subsequent demultiplexing can range from minor data loss that only affects the sequence tags crossing-over, to complete data loss within a sequencing lane or plate. Therefore, researchers should carefully select the most robust sets of sequence tags available to mitigate the potential for data loss while maximizing the likelihood of data recovery in the presence of sequencing, replication, and oligonucleotide synthesis errors.

We designed several large sets of edit metric sequence tags falling into several edit distance categories (Table 2). Although the number of tags within each edit metric set is large, our methodology likely did not yield the largest potential set of edit metric tags for two reasons. First, given sufficient numbers of sampling draws, evolutionary algorithms are likely to produce larger sets of edit metric sequence tags relative to Conway's lexicode algorithm [36], [53]. However, the approach proposed by Ashlock et al. [36], [53] depends on a genetic algorithm that we felt would be slower and more computationally demanding than our lexicode-based approach when evaluating longer sequence tags. Additionally, evolutionary algorithms are sampling-based and unlikely to return identical tag sets across small numbers of runs, which may be problematic depending on the purpose of tag design. Second, our computational shortcut to find tags of edit distance greater than the minimum desired distance increases speed, but it does not return the largest sets of edit metric sequence tags within edit distance categories greater than the minimum input. One can easily maximize the size of each edit metric sequence tag set returned by *EDITTAG* by running the program for only the tag length and minimum edit distance desired. We believe that the shortcut method we used is generally sufficient for most applications, and the amount of computational time this approach saves is large, particularly when evaluating sequence tags over eight nucleotides in length.

Our testing of sequence-tagged PCR primers suggests that the integration of primer design software to the amplicon-tagging process may increase the success of the tagging process by allowing researchers to avoid primers having problematic secondary structures resulting from the placement of sequence tags. Our sample of different primers having integrated tags was relatively small, and we recognize that additional trials using other primers in different organisms will provide a better understanding of the utility of this approach. One specific advantage of this approach is that it is ecumenical because we ligate platform-specific adapters to pooled PCR products after amplification. Thus, researchers can use this approach to obtain sequences from the same PCR primers or product pools on multiple platforms (e.g., 454, Ion Torrent, and Illumina) providing flexibility today and into the future using platforms and adapters that have yet to be developed or released.

Our tests of Nextera and TruSeq-style adapters integrating edit distance tags suggest that both of these approaches were successful and return a number of reads equivalent to indexed adapters from commercial sources. Unfortunately, following the Illumina acquisition of Epicentre, the company modified the structure of Nextera adapters and discontinued the original kits. Thus, the primers that we tested will not work directly with the new Illumina Nextera kits, although edit metric sequence tags could be used to develop an extended set of primers for these new kits.

Pooling of TruSeq-style adapters prior to ligation produced highly variable numbers of reads relative to the equal ratio of adapters we added to the reaction. Variance in the quantification of the input oligonucleotides and pipetting likely contribute to read number variance, but the extent of the variance we observed suggests differences in ligation efficiency among individual adapters, supporting earlier observations of this behavior [54]–[56]. As expected, library preparations directly ligating individual adapters to samples in the standard fashion (T/A ligation) do not show obvious differences in read numbers (Figure S13b). Thus, the sequence tags we designed and integrated to sequencing adapters performed as one would expect.

In practice, researchers often consider “sequencing error” as being comprised of a single error term, identical to the approach we used in the simple models presented in Figure S1. It is important to remember, however, that the error found in sequence reads is actually a composite of several, different sources of error: errors arising during oligonucleotide synthesis, errors arising during the sequence replication process, and errors arising during the sequencing process. Each source of error has a potentially unique bias that contributes to the overall error term. For example, incomplete coupling during oligonucleotide synthesis results in n-1 deletion errors in the final oligonucleotide pool that combine with low-rates of substitution errors on certain sequencing platforms and affect the recovery of sequence tagged DNA reads. Thus, even if a sequencing technology free from deletion errors is used, deletions will still be present in sequence tagged data. The presence of deletions violates the assumptions of certain distance metrics, particularly the Hamming distance, and these violations may corrupt the set of sequence tags used, returning erroneous and potentially misleading data. This example highlights the reasons why it is best to use edit metric-derived sequence tags that are robust to insertions, deletions, and substitutions.

## Conclusions

Our results suggest that all sequence tags should be evaluated prior to their use during MPS because some tags sets do not conform to the metric that maintains the uniqueness of sequence tags in the presence of synthesis, replication, and sequencing errors. We suggest that edit metric sequence tags are superior to tags designed using Hamming distance metrics because edit metric tags are robust to substitution, insertion, and deletion errors, the suite of which likely affect sequence tags at some point during every MPS workflow. Previously, large sets of edit metric sequence tags did not exist for tracking hundreds or thousands of DNA targets during MPS, nor was there a reliable way to generate these edit metric tag sequences. We provide a flexible, computational method to generate large sets of edit metric sequence tags and computer code for incorporating these tags to PCR primers or sequencing adapters. Performance of these edit metric sequence tags during sequencing is equivalent to commercial sources. The tag sets we designed are an improvement over alternatives because they are larger, valid across the set, and more robust to the sources of error affecting recovery of sequence-tagged MPS data. These tag sets may also be used in a variety of configurations to improve the accuracy of tracking and assigning reads to samples [12] and enable concurrent sequencing of hundreds of thousands of samples.

### Availability

Data supporting Figure 4 and Figures S11, S12, S13, S14 are available from Dryad (doi:10.5061/dryad.4m0v8474). All source code and sequence tags generated as part of this manuscript are available from: http://github.com/faircloth-lab/edittag/ under BSD and Creative Commons Attribution licenses. Documentation for the source code is available at http://faircloth-lab.github.com/edittag/. We will provide updated information about validated sequence tags as well as ongoing and future tests of different tag sets and tagging approaches at http://bad-dna.org/tags/.

## Supporting Information

Figure S1 The number of reads returned having errors within sequence tags of different lengths at uniformly distributed sequencing error rates of 1%, 5%, and 18%. The simulation assumes one million reads are returned per sequencing run.
(PDF)

Figure S2 Nucleotide bases can be encoded using a binary representation of each base. For example, we can use a pair of binary values to represent (A) a single nucleotide (a single bit of binary data - 0 or 1 - is insufficient to encode to all nucleotide bases). When encoding sequence tags using their binary representation (B), the binary designation for each base can be arbitrary but must be systematic. The binary representation (C) of each sequence tag is then used in place of the nucleotide representation to compute the desired distance metric and for subsequent sample identification.
(PDF)

Figure S3 Pairwise edit distances between 96 sequence tags described in Supplementary Table 4 of Adey et al. [41]. The minimum expected edit distance of the set is four. The minimum observed edit distance of the set is two.
(PDF)

Figure S4 Pairwise edit distances between 75 sequence tags designed using create_index_sequences.py from Meyer *et al.* [29]. We generated these tags using: ‘python create_index_sequences.py -l <length> -d 3’. The minimum expected edit distance of the set is three. The minimum observed edit distance of the set is two.
(PDF)

Figure S5 Pairwise edit distance comparisons between 24 sequence tags described in Qiu *et al.* [40]. The minimum expected edit distance of the set is three. The minimum observed edit distance of the set is three.
(PDF)

Figure S6 Pairwise edit distance comparisons between 132 sequence tags provided as part of the Roche-454, Inc. multiplex identification (MID) tag set. The minimum expected edit distance of the set is four. The minimum observed edit distance of the set is four.
(PDF)

Figure S7 Pairwise edit distance comparisons between 132 sequence tags provided as part of the Roche-454, Inc. rapid library multiplex identification (RL-MID) tag set. The minimum expected edit distance of the set is four. The minimum observed edit distance of the set is four.
(PDF)

Figure S8 Pairwise edit distance comparisons between 81 sequence tags designed using BARCRAWL [42]. We generated these tags using: ‘barcrawl -l 6 -m 3’. BARCRAWL uses a hybrid approach to account for substitutions and a single deletion that produces sequence tags approximately equal to a minimum edit distance of two, allowing tags to differentiate samples sufficiently in the presence of insertion, substitution, and deletion errors but not allowing for error correction.
(PDF)

Figure S9 Pairwise Hamming distance comparisons between the 48 sequence tags provided as part of the Illumina TruSeq library preparation kits. The tags used within the DNA and RNA kits are a subset of those used within the smallRNA kit. The minimum expected Hamming distance of the set is three. The minimum observed Hamming distance of the set is two.
(PDF)

Figure S10 Agarose gel image of *rbcLa* amplicons generated using fusion-style primers integrating 10 nucleotide, edit distance five sequence tags.
(PDF)

Figure S11 Fold difference in the average number of reads per well (across two plates) for PCR amplicons incorporating sequence tags designed using EDITTAG relative to the average number of reads per plate.
(PDF)

Figure S12 Comparison of the number of reads returned for libraries incorporating adapters having six nucleotide Epicentre Nextera indices or EDITTAG-designed indices.
(PDF)

Figure S13 Comparison of the number of reads returned for libraries prepared using two methods. We prepared the first library (A) by ligating an adapter pool to DNA fragments, and we prepared the second (B) by ligating individual adapters to DNA fragments. Although the numbers of reads are different between runs, note that variance is much higher (>2 orders of magnitude) in (A). Additionally, some adapters (e.g., BFIDT-012) performing poorly in (A) function well in (B).
(PDF)

Figure S14 Comparison of the number of reads returned for 64 libraries incorporating adapters having 10 nucleotide EDITTAG-designed indices versus 12 libraries incorporating Illumina TruSeq adapters. Outliers represent failed enrichments.
(PDF)

Table S1 The counts of sequence tags within commercial and non-commercial sets having a minimum edit distance of three or five.
(PDF)

File S1 Commercial and non-commercial sequence tags in an input format suitable for validation using EDITTAG.
(TXT)

File S2 Ten nucleotide, edit distance five sequence tags that we incorporated into PCR primers and Illumina-style sequencing adapters.
(TXT)

File S3 PCR primers incorporating 10 nucleotide edit metric sequences tags for amplifying *rbcL* in land plants.
(XLSX)

File S4 An extended set of sequencing adapters incorporating edit metric sequence tags for use with the Epicentre Nextera library preparation kit.
(XLSX)

File S5 Illumina-style adapters (*n* = 135) incorporating 10 nucleotide, edit distance five sequence tags. The first 46 of these sequence tags also have an edit distance of three across the first six nucleotides of the index, so they will work in place of TruSeq indexes.
(XLSX)

File S6 All edit distance computations across the tag sets contained within File S1.
(XLSX)

File S7 All edit metric sequence tags we generated as part of this research.
(TXT)
