# Supplementary material for: Not All Sequence Tags Are Created Equal: Designing and Validating Sequence Identification Tags Robust to Indels
Source: PLoS One. 2012 Aug 10;7(8):e42543. doi: 10.1371/journal.pone.0042543 (PMC3416851; doi:10.1371/journal.pone.0042543)

Figure S1. The number of reads returned having errors within sequence tags of different lengths at uniformly distributed sequencing error rates of 1%, 5%, and 18%. The simulation assumes 1 million reads are returned per sequencing run.

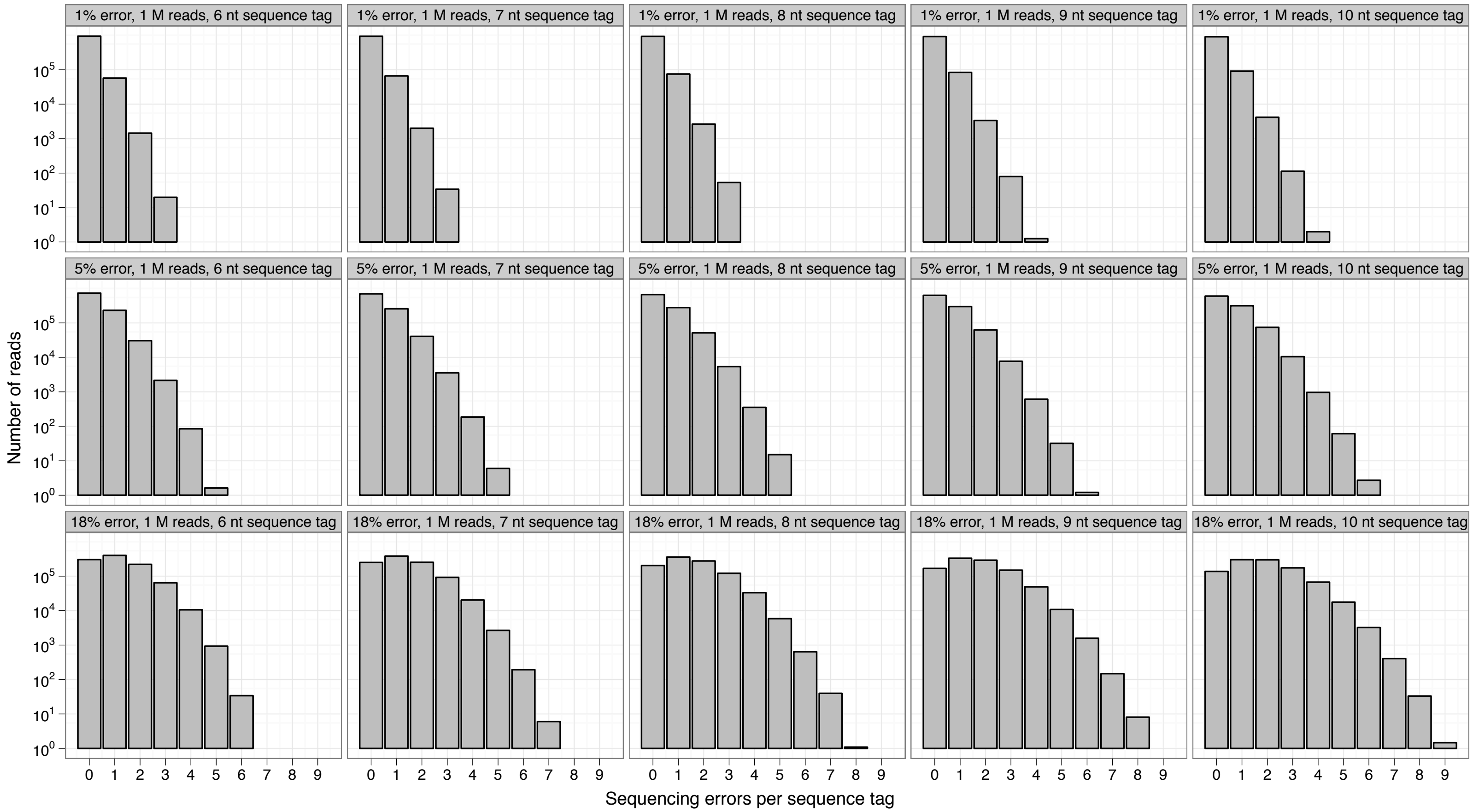

Supplement: Figure S1 — The number of reads returned having errors within sequence tags of different lengths at uniformly distributed sequencing error rates of 1%, 5%, and 18%. The simulation assumes one million reads are returned per sequencing run. (PDF) [file pone.0042543.s001.pdf]
