# Supplementary material for: Not All Sequence Tags Are Created Equal: Designing and Validating Sequence Identification Tags Robust to Indels
Source: PLoS One. 2012 Aug 10;7(8):e42543. doi: 10.1371/journal.pone.0042543 (PMC3416851; doi:10.1371/journal.pone.0042543)

Figure S2. Nucleotide bases can be encoded using a binary representation of each base where a pair of binary values is used to represent (A) a single nucleotide (a single bit of binary data - 0 or 1 - is insufficient to encode to all nucleotide bases). When encoding sequence tags using their binary representation (B), the binary designation for each base can be arbitrary but must be systematic. The binary representation (C) of each sequence tag is then used in place of the nucleotide representation to compute the desired distance metric and for subsequent sample identification.

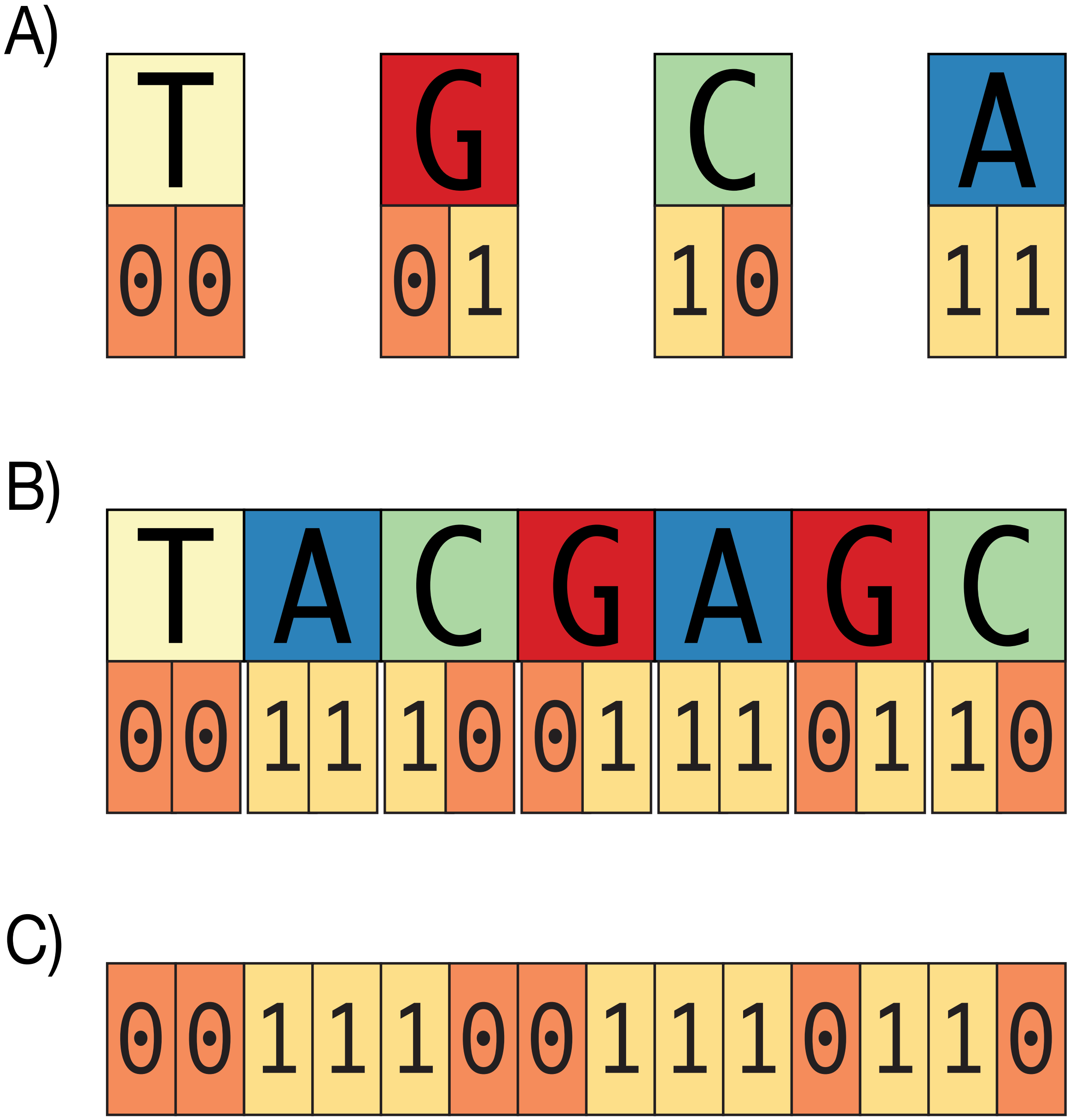

Supplement: Figure S2 — Nucleotide bases can be encoded using a binary representation of each base. For example, we can use a pair of binary values to represent (A) a single nucleotide (a single bit of binary data - 0 or 1 - is insufficient to encode to all nucleotide bases). When encoding sequence tags using their binary representation (B), the binary designation for each base can be arbitrary but must be systematic. The binary representation (C) of each sequence tag is then used in place of the nucleotide representation to compute the desired distance metric and for subsequent sample identification. (PDF) [file pone.0042543.s002.pdf]
