# Supplementary material for: Not All Sequence Tags Are Created Equal: Designing and Validating Sequence Identification Tags Robust to Indels
Source: PLoS One. 2012 Aug 10;7(8):e42543. doi: 10.1371/journal.pone.0042543 (PMC3416851; doi:10.1371/journal.pone.0042543)
Supplement: Figure S4 — Pairwise edit distances between 75 sequence tags designed using create_index_sequences.py from Meyer et al. [29]. We generated these tags using: ‘python create_index_sequences.py -l -d 3’. The minimum expected edit distance of the set is three. The minimum observed edit distance of the set is two. (PDF) [file pone.0042543.s004.pdf]

Figure S4. Pairwise edit distances between 75 sequence tags designed using create_index_sequences.py from Meyer *et al.* 2010. We generated these tags using: `python create_index_sequences.py -l <length> -d 3`. The minimum expected edit distance of the set is three. The minimum observed edit distance of the set is two.

	AACCAG	AACGCA	AACTGC	AAGAAC	AAGCCT	AAGTTA	AATACG	AATCGA	AATGAT	ACCAAT	ACCGTC	ACCTCG	ACGAGG	ACTATA	ACTTAC	AGAAGC	AGACCG	AGAGTA	AGATAT	AGCATG	AGCCGT	AGGCTC	AGTTGG	ATAACT	ATAGAC	ATATTG	ATGGCG	ATTCTT	CAAGAG	CAATTC	CAGATG	CAGCAA	CAGGCC	CATAGC	CCAACG	CCAGGT	CCGCTT	CCGTAG	CCTGAA	CGAATT	CGATCA	CGCAAC	CGCGCT	CGTCAG	CTAAGA	CTCCAT	CTCGGC	CTGCGG	GACCTT	GACGAC	GATTAG	GCATCC	GCCAGC	GCGACT	GCTCAT	GGCCAA	GGCTTC	GGTACC	GTAATC	GTACGT	GTCAAG	GTCTCT	GTTCCG	TACGGT	TACTTG	TATCTC	TCATAA	TCCGAG	TCGCCG	TCTAGT	TCTGCC	TGGAAG	TGGTCC	TTATGC
AACGCA	2																																																																								
AACTGC	3	2																																																																						
AAGAAC	3	3	3																																																																				
AAGCCT	3	3	4	3																																																																		
AAGTTA	4	3	3	3	3																																																																	
AATACG	3	3	3	3	3	4																																																															
AATCGA	3	2	3	4	3	3	2																																																														
AATGAT	3	3	3	3	3	3	3	2																																																												
ACCAAT	3	4	4	3	4	5	4	4	3																																																										
ACCGTC	4	3	3	4	4	4	5	4	4	3																																																								
ACCTCG	3	3	3	5	4	4	3	3	5	3	2																																																						
ACGAGG	3	4	4	3	4	4	3	4	4	3	4	3																																																					
ACTATA	4	4	4	4	5	3	3	3	3	3	3	4	3																																																			
ACTTAC	4	4	3	3	5	3	3	4	3	3	3	3	4	2																																																		
AGAAGC	4	3	3	2	4	5	4	4	4	4	4	5	3	4	4																																																	
AGACCG	2	3	3	3	3	5	3	3	5	5	4	3	3	5	5	3																																																
AGAGTA	4	3	4	3	4	2	4	4	4	4	3	5	3	3	4	3	3																																														
AGATAT	4	5	4	3	4	3	3	4	2	3	5	4	4	3	3	3	3	2																																												
AGCATG	3	4	3	4	3	4	3	4	4	3	3	3	3	3	4	3	3	3	3																																											
AGCCGT	3	4	3	5	2	5	4	3	4	3	2	3	4	4	5	3	2	3	3	3																																										
AGGCTC	4	4	3	3	3	3	5	4	4	5	3	3	4	4	4	3	3	3	4	3	3																																									
AGTTGG	4	5	3	5	5	3	3	3	4	5	5	3	3	4	3	3	3	4	3	3	3	4																																								
ATAACT	4	4	4	3	3	4	3	4	3	3	5	4	4	4	3	3	3	4	3	4	4	4	5																																							
ATAGAC	4	4	4	2	3	4	3	3	3	4	3	5	4	3	3	3	4	3	3	5	5	4	4	2																																						
ATATTG	4	5	3	5	5	3	3	3	4	4	4	3	4	3	4	4	3	3	3	3	5	4	3	3	3																																					
ATGGCG	4	3	4	4	3	4	3	4	4	5	4	3	3	5	4	4	3	4	5	4	3	3	3	3	3	3																																				
ATTCTT	4	5	4	5	3	4	4	3	3	4	4	4	5	3	4	5	4	4	4	4	3	3	4	3	4	3	4																																			
CAAGAG	3	4	4	3	4	4	3	4	3	4	5	5	3	4	5	4	4	4	4	4	5	5	5	5	3	4	4	6																																		
CAATTC	5	4	3	4	4	3	3	4	4	4	4	4	5	4	3	4	5	4	4	5	6	4	4	4	4	3	5	4	3																																			
CAGATG	4	5	4	3	4	3	3	4	3	4	4	4	3	4	5	3	3	3	3	2	4	4	3	5	4	3	4	5	2	3																																				
CAGCAA	3	3	5	3	3	3	5	3	4	3	5	5	5	4	5	4	4	4	4	3	4	4	5	5	4	6	5	5	3	4	3																																			
CAGGCC	5	3	4	3	3	4	4	5	4	6	4	5	4	6	5	4	3	4	5	5	4	2	5	5	4	6	3	5	3	3	3	3																																		
CATAGC	4	4	3	3	4	5	3	3	4	5	4	4	4	4	3	3	4	4	4	4	5	5	4	3	2	4	3	5	3	3	3	4	3																																	
CCAACG	4	4	4	4	5	6	3	4	5	3	4	3	3	4	4	4	3	5	5	4	5	6	5	3	4	4	4	6	3	4	3	4	4	4																																
CCAGGT	4	5	5	5	4	4	5	5	4	3	4	4	3	5	5	4	5	4	4	4	4	4	5	4	4	5	5	5	3	4	3	4	3	4	3																															
CCGCTT	5	4	5	5	3	4	6	5	5	4	3	4	4	4	5	6	5	5	4	4	4	3	5	5	6	5	5	3	5	4	3	3	4	5	4	3																														
CCGTAG	4	4	5	4	5	4	4	5	5	4	3	3	3	4	3	4	5	4	4	4	4	5	4	6	5	4	4	6	3	4	3	3	4	3	3	4	3																													
CCTGAA	5	4	4	4	6	5	5	4	3	4	4	4	4	3	3	5	6	4	5	5	5	6	5	5	4	6	5	5	3	5	4	3	4	4	4	3	4	3																												
CGAATT	6	5	5	4	5	4	5	5	4	3	5	6	4	4	5	3	4	3	3	3	4	4	5	3	5	4	6	4	4	2	3	4	5	4	3	3	3	4	4																											
CGATCA	4	4	5	4	5	4	4	3	4	5	4	4	4	3	5	4	3	3	3	4	5	4	4	4	5	4	5	5	4	3	3	3	4	4	3	4	4	4	4	3																										
CGCAAC	4	4	4	3	5	5	5	5	5	3	4	5	4	5	4	3	4	5	4	3	4	4	5	4	4	6	6	6	4	4	4	2	4	3	2	4	4	4	4	3	3																									
CGCGCT	5	3	4	5	3	5	5	5	4	4	4	4	5	5	4	4	4	4	4	4	3	3	5	4	5	6	4	5	4	5	4	4	3	4	4	3	2	4	4	3	3	3																								
CGTCAG	3	4	5	5	5	4	4	4	4	5	4	3	4	5	4	4	3	5	4	3	4	4	3	5	5	5	4	4	3	5	4	3	4	3	4	4	4	2	3	4	2	3	4																							
CTAAGA	5	4	5	4	5	4	4	4	4	4	6	5	4	3	4	3	5	4	5	5	5	6	5	3	3	4	5	5	2	4	4	4	4	3	3	3	5	4	3	3	3	4	5	3																						
CTCCAT	3	4	5	5	4	6	5	4	4	3	4	5	5	4	4	6	5	6	4	4	3	5	6	4	4	5	5	3	4	5	4	3	5	5	4	4	3	4	4	4	4	3	3	3	4																					
CTCGGC	5	4	3	5	5	6	5	4	5	5	3	4	4	5	4	4	5	5	6	5	4	5	4	5	3	5	3	5	4	4	5	5	3	3	4	3	4	4	4	5	5	3	3	3	3	3																				
CTGCGG	4	5	4	5	4	5	4	4	5	6	5	4	3	5	5	5	4	5	6	4	4	4	4	5	5	4	3	4	4	5	3	3	4	4	4	4	3	3	4	5	5	4	3	3	3	3	2																			
GACCTT	3	4	4	5	3	4	5	4	4	3	3	3	5	4	4	5	3	4	4	4	3	4	5	4	6	4	6	3	5	4	4	4	5	5	5	5	3	5	5	3	4	5	4	5	6	3	5	5																		
GACGAC	3	3	3	3	4	5	4	3	3	4	3	4	3	4	3	3	4	4	4	5	4	4	5	4	3	6	4	6	3	4	4	4	3	4	4	5	5	4	4	5	4	3	4	4	4	4	3	5	3																	
GATTAG	3	5	4	4	5	3	3	4	3	5	6	4	4	4	3	4	4	3	3	4	5	5	3	4	4	3	4	4	3	4	4	4	5	3	5	6	5	3	4	4	3	5	6	3	4	5	5	5	4	3																
GCATCC	5	5	4	5	4	5	4	4	5	5	4	3	5	4	3	4	4	5	4	3	5	4	5	4	4	4	5	5	5	3	5	4	3	3	3	4	5	4	5	4	3	3	4	4	5	4	4	5	4	4	4															
GCCAGC	3	4	3	4	5	6	5	5	6	3	3	4	3	4	4	3	4	5	5	4	3	4	5	5	4	6	5	6	4	4	5	4	4	3	3	3	4	4	4	5	4	3	4	3	4	4	3	5	4	3	4	3														
GCGACT	5	4	5	4	3	5	4	5	4	3	4	4	3	4	4	5	4	4	4	4	4	4	6	3	4	5	4	5	5	4	4	5	4	5	3	4	3	4	4	3	3	3	2	4	5	4	4	5	4	2	5	3	3													
GCTCAT	4	4	5	5	4	5	5	4	3	3	4	4	5	3	3	6	5	5	3	4	3	4	5	4	5	5	6	3	5	5	4	4	6	5	5	4	3	4	3	4	3	4	3	3	4	2	4	4	3	4	3	3	3	3												
GGCCAA	3	4	5	5	4	5	6	4	5	3	5	5	5	4	5	5	4	4	4	4	3	4	5	6	5	6	5	5	5	6	6	3	4	6	4	5	5	5	4	5	4	3	4	3	5	3	5	5	3	3	4	5	3	4	3											
GGCTTC	5	5	3	5	4	4	5	6	6	5	3	4	6	4	3	4	5	4	4	3	4	2	4	6	5	4	5	4	6	3	5	5	4	5	5	6	3	5	5	4	4	3	4	5	6	5	4	5	3	3	4	3	3	4	4	3										
GGTACC	5	5	5	4	5	5	3	5	5	5	5	5	5	4	4	3	3	4	4	4	5	4	4	4	4	5	5	5	6	5	5	5	4	3	4	6	6	4	5	4	4	3	4	4	5	5	5	5	4	4	4	3	3	3	4	4	3									
GTAATC	5	5	4	4	5	5	4	4	4	4	4	5	5	4	4	3	5	4	4	4	5	4	5	3	3	3	5	4	5	3	4	5	5	4	4	5	5	5	5	3	4	3	5	4	3	4	4	5	4	4	4	3	3	4	3	5	3	3								
GTACGT	5	4	4	5	4	5	3	4	4	5	4	5	5	5	5	4	4	4	4	4	3	5	4	3	4	4	4	3	5	5	5	5	5	4	4	3	4	4	6	4	5	5	4	4	3	3	4	3	3	3	4	4	4	3	3	4	4	3	3							
GTCAAG	3	5	5	4	6	5	4	4	5	3	5	4	4	5	5	4	4	5	5	3	5	5	4	4	4	4	4	5	4	5	4	4	5	4	3	4	6	4	4	5	4	3	5	2	3	3	4	4	4	3	3	4	3	4	3	3	4	4	3	3						
GTCTCT	5	4	4	6	4	5	5	5	5	4	4	3	6	4	5	5	4	5	4	5	4	3	4	3	5	4	4	3	6	5	6	6	5	5	5	5	4	5	5	4	4	5	3	4	5	3	4	5	3	4	4	3	4	3	2	4	3	4	3	3	3					
GTTCCG	4	5	5	6	4	5	3	4	5	6	5	4	5	5	4	5	3	6	5	5	4	5	3	4	5	4	3	3	5	5	5	5	5	5	4	6	5	5	5	6	4	5	5	3	5	4	4	3	4	5	3	3	4	4	3	4	4	3	4	3	3	3				
TACGGT	4	3	3	5	4	4	4	4	3	4	3	5	3	4	5	5	4	4	5	5	3	4	5	4	4	4	4	4	4	5	4	5	4	4	5	3	4	5	5	5	5	5	3	5	4	4	3	3	3	3	5	6	4	4	5	5	5	5	5	2	5	4	4			
TACTTG	3	4	3	5	4	3	4	4	5	5	4	3	4	3	3	5	4	5	5	3	5	5	3	4	5	2	5	3	4	3	3	5	5	4	5	5	4	4	5	4	5	5	5	5	5	4	5	4	3	4	3	5	5	5	5	5	3	5	4	3	4	4	4	3		
TATCTC	4	4	3	4	4	4	4	3	4	5	3	3	6	4	4	5	5	5	4	4	5	3	5	3	4	3	4	3	5	3	4	4	4	3	5	5	4	6	5	5	4	5	5	4	5	5	4	5	3	4	4	3	5	5	4	5	4	4	3	4	5	3	4	4	3	
TCATAA	5	5	5	4	6	4	4	4	4	4	5	4	5	3	3	5	5	4	3	4	6	6	5	4	4	4	6	5	4	4	4	3	5	3	4	4	5	3	3	5	3	4	6	4	4	4	5	6	5	5	4	3	5	5	4	4	5	5	4	5	3	4	6	5	4	4							
TCCGAG	3	4	5	5	6	6	5	4	4	3	3	3	3	5	4	5	5	5	5	4	4	6	5	6	4	5	4	5	3	6	3	4	5	4	3	4	4	2	3	5	5	4	4	4	4	3	4	3	5	3	4	5	3	4	4	4	5	6	6	4	3	5	4	3	3	5	3						
TCGCCG	4	4	5	5	3	5	4	5	6	5	4	3	3	5	5	5	3	6	6	4	3	4	5	5	4	5	3	5	5	6	4	4	4	5	3	5	3	3	5	5	4	4	3	3	5	4	3	3	5	4	5	4	4	3	4	4	5	5	5	5	4	4	3	4	4	4	4	3					
TCTAGT	4	6	4	5	5	5	4	4	4	3	4	4	3	3	4	4	6	4	4	5	4	6	4	4	4	4	5	4	4	4	4	5	5	3	4	3	4	3	4	4	5	5	4	4	3	3	4	4	5	5	4	5	3	3	3	5	5	4	4	3	3	3	5	3	3	4	3	3	4				
TCTGCC	6	4	3	5	4	6	4	5	4	5	3	4	5	4	3	5	5	5	6	5	5	4	5	5	4	5	4	5	5	5	5	4	3	4	4	4	4	5	3	6	5	4	4	5	5	4	3	3	6	4	5	3	4	4	4	5	4	3	5	5	5	3	4	4	4	3	4	3	2	3				
TGGAAG	4	5	6	3	5	5	4	5	5	4	6	5	3	5	5	3	4	4	4	3	5	4	4	5	4	5	3	6	4	6	3	4	5	5	4	5	5	3	4	4	5	3	5	3	4	5	5	4	6	5	4	5	4	4	5	4	5	4	4	4	3	6	5	5	4	5	4	3	3	4	5			
TGGTCC	6	5	4	4	4	4	5	5	5	6	4	4	5	6	4	4	4	4	4	5	5	3	4	5	4	5	3	5	6	4	5	5	3	5	5	5	5	4	6	5	3	4	4	4	6	5	4	5	5	5	5	3	5	4	4	4	3	2	4	5	5	4	3	4	4	3	4	5	3	5	3	3		
TTATGC	6	4	3	5	5	5	4	4	4	6	5	5	5	4	4	3	5	5	4	4	5	5	4	4	3	3	4	4	5	3	4	5	4	3	5	4	6	5	5	5	4	5	5	5	3	5	3	4	6	4	4	3	4	6	5	6	4	4	3	3	4	4	4	4	4	3	3	5	5	3	3	4	3	
TTGATT	6	6	6	4	4	4	5	5	3	4	5	6	4	4	5	5	5	4	3	4	5	4	5	3	4	3	4	3	5	4	3	5	5	5	5	5	3	5	4	3	4	5	5	5	4	4	5	4	4	5	4	5	5	3	4	6	4	5	3	4	4	4	5	4	4	4	4	4	4	3	4	3	4	3
