# Supplementary material for: Not All Sequence Tags Are Created Equal: Designing and Validating Sequence Identification Tags Robust to Indels
Source: PLoS One. 2012 Aug 10;7(8):e42543. doi: 10.1371/journal.pone.0042543 (PMC3416851; doi:10.1371/journal.pone.0042543)
Supplement: Figure S5 — Pairwise edit distance comparisons between 24 sequence tags described in Qiu et al. [40]. The minimum expected edit distance of the set is three. The minimum observed edit distance of the set is three. (PDF) [file pone.0042543.s005.pdf]

Figure S5. Pairwise edit distance comparisons between 24 sequence tags described in Qiu *et al.* 2003. The minimum expected edit distance of the set is three. The minimum observed edit distance of the set is three.

	ATACGC	ACTGGC	CACAGC	TAACCC	CAGGCG	AGGTAC	TGAGCG	GACCAC	AATCGG	CTAAGG	GAAGAG	AGTGAG	GTGGAC	GTCACC	CGTCCA	GATGCC	AAGACC	GCCTCA	CTAGCC	TACGGA
ACTGGC	3																			
CACAGC	3	3																		
TAACCC	3	5	4																	
CAGGCG	4	3	3	4																
AGGTAC	4	4	5	5	4															
TGAGCG	4	4	4	3	3	5														
GACCAC	4	4	3	3	5	4	4													
AATCGG	3	3	4	4	4	5	4	4												
CTAAGG	3	4	3	4	3	6	3	6	4											
GAAGAG	5	5	4	4	3	4	3	3	4	3										
AGTGAG	4	3	5	6	3	3	3	5	3	4	3									
GTGGAC	4	3	5	5	4	3	4	3	5	5	3	3								
GTCACC	3	5	3	3	5	4	4	3	5	4	5	4	3							
CGTCCA	5	5	5	4	4	4	4	3	4	5	5	4	4	3						
GATGCC	3	3	4	3	3	4	3	3	4	5	3	4	3	3	4					
AAGACC	3	4	3	3	3	3	4	4	4	5	3	4	4	3	4	3				
GCCTCA	5	5	5	5	5	5	5	3	5	5	5	5	5	3	3	4	5			
CTAGCC	3	3	3	3	3	5	3	4	5	3	4	5	3	3	4	3	3	5		
TACGGA	3	3	3	4	4	4	4	4	3	3	4	4	4	5	5	4	5	4	4	
GCAGGA	4	3	4	5	3	4	4	4	5	4	3	4	3	4	5	4	5	3	4	3
