# Supplementary material for: Not All Sequence Tags Are Created Equal: Designing and Validating Sequence Identification Tags Robust to Indels
Source: PLoS One. 2012 Aug 10;7(8):e42543. doi: 10.1371/journal.pone.0042543 (PMC3416851; doi:10.1371/journal.pone.0042543)
Supplement: Figure S6 — Pairwise edit distance comparisons between 132 sequence tags provided as part of the Roche-454, Inc. multiplex identification (MID) tag set. The minimum expected edit distance of the set is four. The minimum observed edit distance of the set is four. (PDF) [file pone.0042543.s006.pdf]

Figure S6: Pairwise edit distance comparisons between 132 sequence tags provided as part of the Roche-454, Inc. multiplex identification (MID) tag set. The minimum expected edit distance of the set is four. The minimum observed edit distance of the set is four.

		MID-1 ACGAGTGCGT	MID-2 ACGCTCGACA	MID-3 AGACGCACTC	MID-4 AGCACTGTAG	MID-5 ATCAGACACG	MID-6 ATATCGCGAG	MID-7 CGTGTCTCTA	MID-8 CTCGCGTGTC	MID-10 TCTCTATGCG	MID-11 TGATACGTCT	MID-13 CATAGTAGTG	MID-14 CGAGAGATAC	MID-15 ATACGACGTA	MID-16 TCACGTACTA	MID-17 CGTCTAGTAC
MID-2	ACGCTCGACA	6														
MID-3	AGACGCACTC	6	6													
MID-4	AGCACTGTAG	6	6	6												
MID-5	ATCAGACACG	6	6	6	6											
MID-6	ATATCGCGAG	6	6	6	6	6										
MID-7	CGTGTCTCTA	6	6	6	6	8	6									
MID-8	CTCGCGTGTC	6	6	6	6	7	6	6								
MID-10	TCTCTATGCG	6	6	8	6	6	6	7	6							
MID-11	TGATACGTCT	6	6	6	6	6	7	6	7	6						
MID-13	CATAGTAGTG	6	8	6	6	6	6	6	6	6	6					
MID-14	CGAGAGATAC	6	6	6	6	6	7	6	6	8	6	6				
MID-15	ATACGACGTA	6	6	4	5	5	4	7	6	7	5	5	6			
MID-16	TCACGTACTA	6	6	4	6	6	7	5	5	6	5	5	7	4		
MID-17	CGTCTAGTAC	7	5	6	5	6	7	5	5	5	6	5	4	6	6	
MID-18	TCTACGTAGC	6	7	6	5	7	6	8	5	4	5	5	6	5	4	4
MID-19	TGTACTACTC	7	7	4	6	7	8	5	7	5	4	5	7	5	4	4
MID-20	ACGACTACAG	4	4	5	4	4	6	7	8	6	7	6	6	6	5	5
MID-21	CGTAGACTAG	7	5	6	5	5	6	5	7	7	6	4	4	5	6	4
MID-22	TACGAGTATG	4	7	6	6	6	5	7	5	6	6	4	6	4	5	6
MID-23	TACTCTCGTG	6	5	6	5	7	4	6	5	4	6	5	9	5	6	6
MID-24	TAGAGACGAG	6	7	6	5	5	4	8	7	6	5	5	5	5	6	8
MID-25	TCGTCGCTCG	6	5	6	7	6	5	5	5	4	5	6	7	6	6	6
MID-26	ACATACGCGT	4	6	6	7	4	4	7	6	5	4	5	8	4	7	7
MID-27	ACGCGAGTAT	4	5	6	5	7	6	6	5	7	6	6	4	4	5	4
MID-28	ACTACTATGT	4	7	7	5	6	7	7	6	4	6	5	7	4	6	6
MID-29	ACTGTACAGT	4	6	7	6	5	6	6	7	6	6	6	7	6	6	6
MID-30	AGACTATACT	6	5	4	4	6	7	6	8	5	5	6	5	6	5	4
MID-31	AGCGTCGTCT	5	4	5	5	6	7	4	5	7	4	6	7	5	6	6
MID-32	AGTACGCTAT	6	6	4	4	7	4	5	6	7	4	6	6	4	6	6
MID-33	ATAGAGTACT	4	7	5	6	4	6	7	6	7	5	5	4	4	5	5
MID-34	CACGCTACGT	4	5	5	6	6	6	6	5	6	6	5	6	5	4	6
