# Supplementary material for: Not All Sequence Tags Are Created Equal: Designing and Validating Sequence Identification Tags Robust to Indels
Source: PLoS One. 2012 Aug 10;7(8):e42543. doi: 10.1371/journal.pone.0042543 (PMC3416851; doi:10.1371/journal.pone.0042543)

Figure S7. Pairwise edit distance comparisons between 132 sequence tags provided as part of the Roche-454, Inc. rapid library multiplex identification (RL-MID) tag set. The minimum expected edit distance of the set is four. The minimum observed edit distance of the set is four.

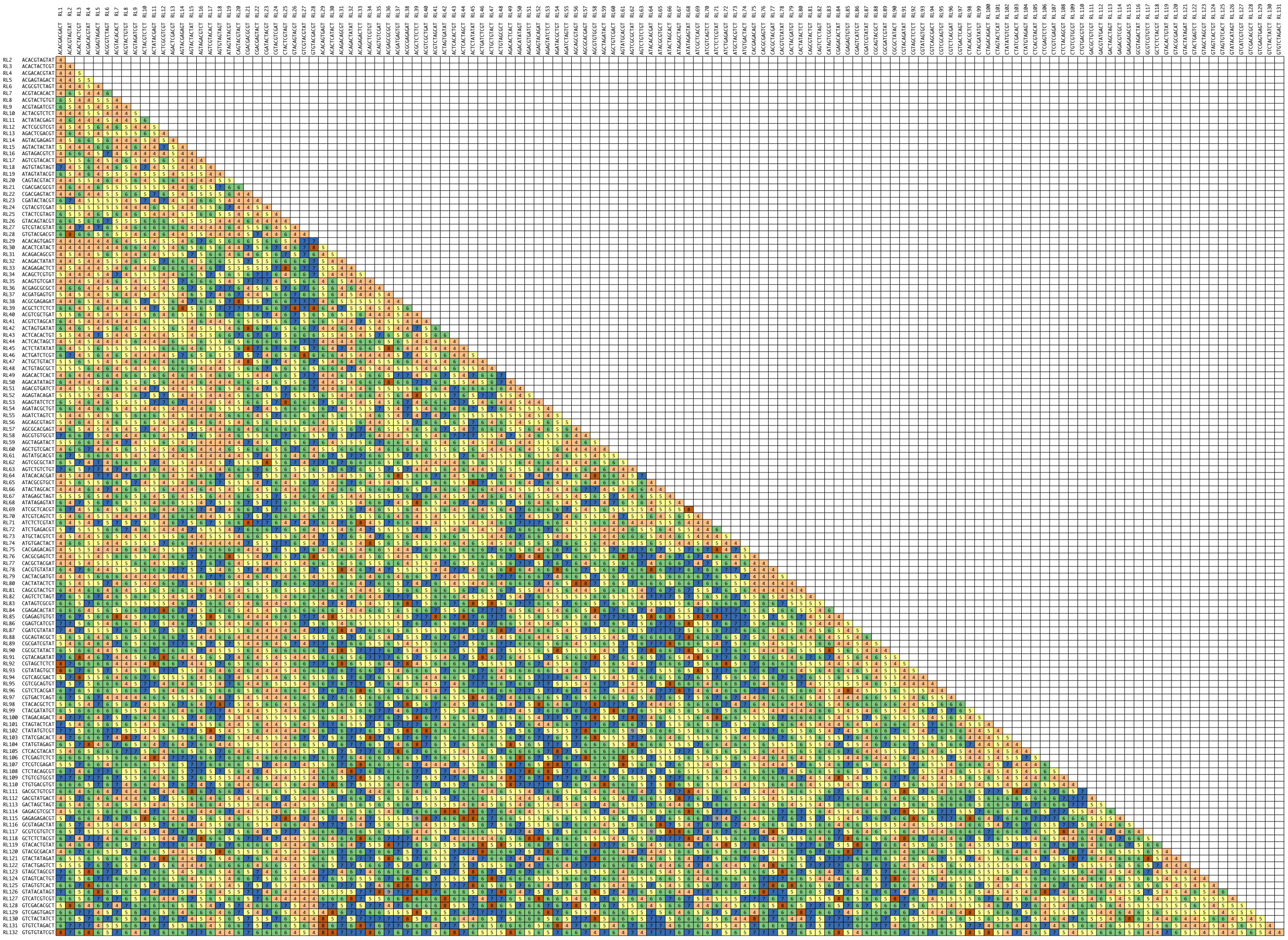

Supplement: Figure S7 — Pairwise edit distance comparisons between 132 sequence tags provided as part of the Roche-454, Inc. rapid library multiplex identification (RL-MID) tag set. The minimum expected edit distance of the set is four. The minimum observed edit distance of the set is four. (PDF) [file pone.0042543.s007.pdf]
