# Supplementary material for: Not All Sequence Tags Are Created Equal: Designing and Validating Sequence Identification Tags Robust to Indels
Source: PLoS One. 2012 Aug 10;7(8):e42543. doi: 10.1371/journal.pone.0042543 (PMC3416851; doi:10.1371/journal.pone.0042543)
Supplement: Figure S8 — Pairwise edit distance comparisons between 81 sequence tags designed using BARCRAWL [42]. We generated these tags using: ‘barcrawl -l 6 -m 3’. BARCRAWL uses a hybrid approach to account for substitutions and a single deletion that produces sequence tags approximately equal to a minimum edit distance of two, allowing tags to differentiate samples sufficiently in the presence of insertion, substitution, and deletion errors but not allowing for error correction. (PDF) [file pone.0042543.s008.pdf]

Figure S8: Pairwise edit distance comparisons between 81 sequence tags designed using barcrawl Frank 2009. We generated these tags using: `barcrawl -l 6 -m 3`. Barcrawl uses a hybrid approach to account for substitutions and a single deletion that produces sequence tags approximately equal to a minimum edit distance of two, allowing tags to differentiate samples sufficiently in the presence of insertion, substitution, and deletion errors but not allowing for error correction.

	TTATCC	TTAAGG	TTCTTG	TTCAAC	TTCCGT	TTCGCA	TTGTGA	TTGACT	TTGGTC	TATTCG	TATAGC	TAACTC	TACCAA	TACGTT	TAGTAC	TAGATG	TCTACA	TCTCGG	TCTGAT	TCATGT	TCAGCG	TCGCTT	TGTCTA	TGTGCC	TGACAG	TGAGGA	TGCACG	ATTCTC	ATTGAG	ATACCT	ATCTGC	ATGCAA	AATCCA	AATGGT	AACTAG	AACACT	AAGAGA	AAGGCC	ACTTAC	ACATTG	ACAACC	ACACGA	ACCTCA	ACCAGG	ACGAAT	ACGGTA	AGTTGG	AGTCAT	AGAAGT	AGCCTG	AGCGAC	AGGTTC	CTTATG	CTACGC	CTAGAA	CTCTCT	CTCCAG	CATCTT	CATGAC	CAAGTG	CACATA	CCTTGA	CCAATT	CCGTAG	CGTACT	CGATTA	CGGTGT	CGGAAC	GTTAGT	GTAACA	GTGTAT	GTGCTG	GATAAG	GAATCT	GAACGG	GACTGA	GCTATC	GCTCAA	GGATGC	GGACTT	GGCAAT
TTAAGG	3
TTCTTG	3	3
TTCAAC	3	3	3
TTCCGT	4	3	3	3
TTCGCA	3	4	3	3	3
TTGTGA	3	3	3	4	3	3
TTGACT	3	3	4	3	3	3	3
TTGGTC	3	4	3	3	3	3	3	3
TATTCG	3	4	3	5	3	4	4	4	4
TATAGC	3	3	4	3	4	3	4	4	3	3
TAACTC	3	4	4	4	4	4	5	3	3	3	3
TACCAA	4	5	4	3	3	3	4	5	5	4	4	3
TACGTT	5	4	3	4	3	3	4	4	3	4	4	3	3
TAGTAC	3	5	4	3	5	4	3	3	3	3	2	3	3	3
TAGATG	4	3	3	4	5	5	3	3	3	3	3	3	4	3	3
TCTACA	3	4	4	3	4	3	4	3	5	3	3	4	3	5	3	4
TCTCGG	4	3	3	4	3	3	4	5	4	3	3	4	4	4	5	4	3
TCTGAT	4	4	4	4	3	4	3	2	3	4	4	5	4	3	4	3	3	3
TCATGT	3	3	3	4	3	5	3	4	4	4	3	4	5	3	4	3	4	3	2
TCAGCG	3	3	4	3	4	3	4	4	4	3	3	4	4	4	4	3	3	3	3	3
TCGCTT	5	5	3	5	3	3	4	3	3	5	5	3	4	2	4	3	4	3	3	3	3
TGTCTA	4	5	3	3	4	3	3	3	4	4	4	3	3	4	4	4	3	3	4	4	5	3
TGTGCC	3	5	5	4	4	3	3	3	3	3	3	4	5	4	3	4	3	4	3	4	3	4	3
TGACAG	4	3	4	4	4	4	4	3	5	4	3	3	3	4	4	3	3	3	4	4	3	4	3	4
TGAGGA	4	3	5	5	4	3	3	4	4	5	4	4	4	4	3	3	4	4	4	3	3	5	3	3	3
TGCACG	4	3	3	3	3	3	5	3	4	3	4	4	4	4	3	3	3	3	5	3	2	4	4	3	2	3
ATTCTC	3	5	3	3	4	3	4	4	3	3	4	3	5	4	4	5	4	4	5	5	5	4	3	4	5	6	4
ATTGAG	5	3	4	4	4	4	3	3	4	3	4	6	5	5	4	3	5	4	3	5	4	5	5	4	4	4	4	3
ATACCT	3	4	4	4	3	4	5	3	5	4	3	3	3	3	4	4	4	5	5	4	4	4	4	4	4	5	4	3	4
ATCTGC	3	4	3	3	3	3	3	4	4	4	3	3	5	5	4	4	3	3	3	3	3	4	4	3	5	5	5	2	3	4
ATGCAA	5	5	5	3	4	3	3	4	4	4	4	5	3	5	4	5	4	5	4	5	5	4	3	4	3	4	3	3	3	3	4
AATCCA	3	6	5	4	4	3	5	5	6	3	4	4	3	5	4	5	3	4	5	5	5	5	3	4	5	5	5	3	4	3	4	3
AATGGT	5	4	5	6	4	5	4	4	3	4	3	5	5	3	5	4	5	4	3	3	5	5	5	4	5	4	6	4	3	4	4	4	3
AACTAG	5	5	3	4	5	5	5	5	6	3	3	3	3	4	3	4	4	4	4	5	5	5	4	6	3	5	4	4	3	4	3	4	3	3
AACACT	5	5	5	4	4	4	6	3	6	4	4	3	4	3	4	4	4	5	5	4	4	4	5	5	4	6	3	4	5	2	4	4	3	3	3
AAGAGA	6	4	6	5	5	5	3	4	5	5	3	5	4	5	4	3	4	5	5	5	4	5	5	5	4	3	5	5	3	5	4	3	3	3	3	3
AAGGCC	4	4	6	5	6	4	5	4	3	4	4	4	5	4	3	4	5	6	5	5	4	5	5	3	5	4	5	4	4	4	4	4	3	3	4	3	3
ACTTAC	4	5	4	4	5	5	5	4	5	4	4	4	4	4	3	5	3	4	3	4	5	5	4	4	5	6	5	3	3	4	3	4	4	4	3	4	5	4
ACATTG	4	4	3	5	5	6	5	5	5	3	4	4	5	3	5	3	5	4	5	3	3	4	5	6	4	5	4	4	4	4	4	5	4	3	3	3	4	5	3
ACAACC	3	4	6	3	6	5	6	4	5	5	4	3	4	5	4	5	3	5	5	4	3	5	6	4	5	5	4	4	5	3	4	4	3	5	4	3	4	3	3	3
ACACGA	5	4	5	5	4	4	4	5	6	4	4	4	3	4	5	4	3	3	4	3	3	4	4	6	4	3	3	4	4	3	4	3	3	4	3	3	3	4	4	3	3
ACCTCA	4	6	4	5	5	3	4	5	5	4	5	3	3	4	5	5	3	4	4	4	4	4	4	5	5	5	4	3	4	4	3	4	3	5	3	3	4	4	3	3	3	3
ACCAGG	6	3	4	4	4	5	5	5	6	5	4	5	3	5	5	4	4	3	5	4	3	5	6	6	4	4	3	5	4	5	3	4	4	4	3	3	3	4	4	3	3	3	3
ACGAAT	6	5	6	4	5	4	5	3	5	6	5	5	3	3	4	4	4	5	3	4	5	3	5	6	4	5	5	5	4	4	5	3	5	4	4	3	3	4	3	3	3	4	4	3
ACGGTA	6	5	5	6	4	4	4	5	3	6	5	5	4	3	4	4	4	5	4	4	4	3	4	5	5	4	6	4	4	5	4	3	4	3	4	4	3	3	3	3	4	3	3	4	3
AGTTGG	5	4	4	6	5	5	4	5	4	3	4	5	6	4	4	3	5	3	5	4	5	5	4	4	4	4	4	4	2	5	3	5	4	3	3	5	4	5	3	3	5	4	4	3	5	4
AGTCAT	5	5	4	4	4	5	4	4	5	4	5	5	4	4	3	4	4	4	3	4	5	4	3	4	3	5	4	3	3	3	4	3	3	3	4	3	4	4	3	4	5	4	3	4	3	4	3
AGAAGT	5	3	6	5	4	6	5	4	5	5	4	4	5	4	4	3	5	5	5	3	5	5	5	5	3	3	4	5	4	3	4	4	5	3	4	3	3	4	5	4	3	3	5	3	2	4	3	3
AGCCTG	5	5	3	5	4	5	5	4	5	4	6	4	4	4	5	3	6	4	5	4	4	4	3	5	3	5	3	3	4	3	3	4	4	5	3	3	5	4	4	3	5	4	3	3	4	4	3	3	4
AGCGAC	5	6	5	3	5	4	4	4	4	5	4	4	4	4	3	5	4	5	4	6	4	5	4	3	4	4	3	4	3	5	3	3	4	4	3	3	3	3	3	5	3	3	4	4	3	4	4	3	4	3
AGGTTC	4	6	4	5	6	6	4	5	3	4	5	4	6	3	3	4	5	6	5	5	6	4	4	4	5	5	5	3	5	5	3	4	4	4	4	5	4	3	3	3	4	5	4	5	4	3	3	4	4	3	3
CTTATG	3	3	3	4	4	5	3	4	4	4	4	4	6	5	5	3	4	4	3	3	4	5	4	5	4	4	4	3	3	5	4	5	5	5	4	5	5	6	3	3	5	5	5	4	4	5	4	4	5	4	6	5
CTACGC	3	3	5	4	3	3	4	4	4	4	3	3	4	3	4	5	3	3	5	4	4	4	5	4	4	4	4	3	5	3	3	4	5	5	4	4	5	4	4	5	3	3	5	4	5	5	5	5	4	5	4	5	3
CTAGAA	4	4	5	4	5	3	4	4	4	5	4	5	3	4	3	3	3	4	3	5	4	5	5	5	4	3	5	5	3	4	5	3	5	5	4	5	3	5	4	5	5	4	5	4	4	4	5	5	4	6	4	6	4	3
CTCTCT	3	5	3	4	3	3	4	3	4	4	5	4	5	4	5	5	3	3	3	3	4	3	3	4	5	6	4	3	5	3	3	5	4	5	4	3	6	5	4	5	5	5	3	5	5	5	5	4	5	4	5	5	3	3	4
CTCCAG	5	4	3	3	3	3	5	5	5	4	4	5	3	5	5	4	3	3	4	4	4	4	4	5	3	5	3	4	3	4	4	3	3	6	3	5	5	6	4	5	5	4	4	3	5	5	5	4	5	3	4	6	3	3	3	3
CATCTT	4	6	3	5	4	5	5	5	5	4	4	3	4	3	5	4	5	4	4	3	4	3	3	5	5	6	5	3	5	3	3	4	3	3	4	4	5	5	4	3	4	4	4	5	5	5	5	3	5	4	5	4	3	4	5	2	4
CATGAC	4	5	5	4	6	5	4	3	4	4	3	4	4	4	3	4	4	5	3	3	4	6	5	3	4	4	4	4	3	4	4	3	4	3	4	4	4	3	3	4	4	3	4	5	4	5	5	4	5	5	3	4	4	3	3	4	4	3
CAAGTG	5	3	4	5	5	5	4	5	4	4	4	3	5	3	4	3	5	4	5	3	3	5	5	5	4	4	5	5	4	5	4	5	5	4	3	4	3	4	5	3	4	4	5	3	4	4	4	5	3	4	5	4	3	4	3	5	4	3	3
CACATA	5	5	4	4	5	4	5	5	5	5	4	4	3	3	4	3	4	5	4	4	5	4	4	6	4	5	4	5	5	4	5	4	4	5	3	3	3	5	3	3	4	3	3	4	4	3	5	4	5	4	5	5	3	4	3	4	4	3	4	3
CCTTGA	5	5	3	5	5	4	3	4	5	4	4	6	5	5	5	5	3	3	3	3	5	4	4	5	6	4	5	5	3	6	4	5	4	4	4	6	4	6	3	3	5	3	3	4	5	4	3	5	5	4	5	5	3	4	3	4	4	4	3	4	3
CCAATT	5	4	5	4	5	6	6	4	5	5	5	4	4	4	5	4	4	5	4	3	4	3	5	6	5	5	5	5	6	4	6	5	5	4	5	4	5	6	5	3	3	4	5	4	3	4	5	5	3	5	5	5	3	4	4	4	4	3	5	3	3	4
CCGTAG	5	5	4	5	4	4	4	5	5	4	4	6	5	4	3	4	4	4	4	4	4	4	5	5	4	5	4	6	4	6	5	4	6	5	3	6	4	5	3	3	5	4	4	4	3	3	4	4	4	4	4	4	3	4	4	4	3	5	4	3	3	3	4
CGTACT	4	5	5	5	4	5	5	3	5	4	4	4	5	4	3	5	3	5	4	4	5	4	3	3	4	5	3	4	5	3	5	5	4	4	5	3	5	5	3	5	4	5	4	5	3	4	4	3	3	4	4	4	3	3	4	3	4	3	3	4	4	4	3	3
CGATTA	4	5	4	6	6	5	4	4	5	4	5	4	5	4	4	4	4	6	4	4	5	3	3	5	4	3	5	5	4	5	5	5	4	5	4	5	4	6	4	3	5	4	4	5	3	3	4	4	4	4	5	3	4	4	3	4	5	3	3	3	3	3	3	4	3
CGGTGT	5	5	5	6	4	6	3	4	4	5	5	6	6	4	4	4	6	5	4	3	4	3	4	4	5	4	5	5	5	5	4	5	6	4	5	5	4	5	5	4	6	5	5	5	4	3	3	4	3	4	5	3	4	4	5	3	5	4	4	3	5	3	4	3	3	3
CGGAAC	5	5	6	3	6	5	5	4	4	6	4	5	5	6	3	4	5	6	4	6	5	5	5	4	4	4	4	5	5	5	5	4	6	6	5	5	4	4	4	6	4	5	5	5	3	3	5	4	4	5	3	3	4	4	3	5	4	5	3	5	4	5	4	3	3	4	3
GTTAGT	4	3	4	4	3	4	4	3	3	4	3	4	6	4	4	4	4	4	4	3	4	5	4	5	4	4	5	4	3	4	4	5	5	3	4	4	4	6	4	5	5	5	6	4	4	5	3	3	3	5	5	5	3	4	4	4	4	4	5	4	5	4	4	4	3	4	3	5
GTAACA	3	3	5	3	5	3	4	3	5	4	4	3	3	5	4	4	3	5	5	5	4	5	4	4	3	4	4	5	5	3	5	4	4	6	4	4	4	5	4	5	3	4	4	5	5	5	5	4	4	6	4	5	4	4	3	4	4	5	4	5	4	5	4	5	3	4	6	3	3
GTGTAT	4	5	4	4	4	4	3	3	4	5	4	5	5	4	3	4	4	5	3	4	6	4	3	4	4	4	4	4	4	4	4	3	5	4	4	5	5	5	4	5	6	6	5	6	3	4	4	3	4	5	4	4	3	5	4	3	4	4	4	5	5	5	5	3	3	4	3	4	2	4
GTGCTG	5	4	3	5	4	4	3	3	3	4	5	4	5	4	5	3	5	4	4	4	4	3	3	3	3	4	3	3	4	4	3	3	5	4	4	5	5	5	6	4	6	5	5	5	5	4	4	4	5	3	5	4	3	4	5	4	3	4	5	4	5	5	5	4	3	5	4	5	4	4	3
GATAAG	5	3	5	4	5	6	5	5	6	3	3	5	4	5	4	3	4	4	4	5	5	6	5	5	3	4	4	5	3	4	4	4	4	4	3	4	4	5	4	4	4	4	5	4	4	5	4	4	3	5	5	5	3	4	4	6	4	4	3	4	4	5	5	4	4	3	5	4	3	3	4	4
GAATCT	3	5	5	5	5	5	5	4	5	3	4	3	5	4	4	4	5	5	5	3	4	5	4	5	5	5	4	3	5	3	4	5	3	3	4	3	5	4	5	4	4	5	4	6	4	5	5	3	3	4	5	4	5	5	5	3	6	3	5	4	4	5	3	5	3	4	4	4	4	3	3	4	4
GAACGG	5	3	5	5	4	5	5	5	6	4	4	3	4	4	5	4	5	3	6	4	4	5	5	5	3	4	3	5	4	4	4	5	4	3	3	4	4	4	6	4	4	3	5	3	5	4	4	5	3	4	4	6	5	3	5	6	4	4	5	3	5	5	5	5	4	5	5	4	4	3	5	3	3	3
GACTGA	5	5	4	5	4	4	3	4	6	4	4	4	3	4	4	4	4	4	3	4	5	5	3	5	3	3	4	5	3	5	3	4	4	4	3	4	3	5	3	4	5	3	3	4	4	3	4	4	4	3	4	5	5	4	4	4	5	4	3	4	3	3	5	5	4	3	4	5	4	4	4	3	3	3	3
GCTATC	3	5	4	4	5	5	5	5	4	4	3	4	5	5	4	4	3	4	3	4	4	4	4	4	5	6	4	3	5	4	4	5	5	5	4	5	5	5	3	4	3	5	4	4	4	4	5	4	5	4	3	3	3	3	4	3	5	4	4	4	4	4	3	4	4	4	4	4	3	3	3	4	3	3	5	4
GCTCAA	5	5	5	3	5	4	5	5	5	5	5	5	3	6	5	6	3	3	3	5	5	4	3	5	4	5	4	4	4	5	5	3	3	5	4	5	5	6	3	5	5	3	3	4	4	4	5	3	6	4	4	5	4	4	3	4	3	4	4	6	3	3	4	4	5	4	5	4	4	3	4	4	3	4	4	3	3
GGATGC	3	4	5	5	5	5	4	5	4	4	3	4	6	5	4	3	5	5	5	3	4	5	5	3	4	3	4	5	5	5	3	4	4	4	5	5	4	4	4	4	4	4	5	5	5	5	3	5	3	4	4	3	4	3	5	5	6	5	3	4	5	4	5	5	4	3	3	3	4	4	4	3	4	3	3	3	3	5
GGACTT	5	5	4	6	4	6	6	3	5	5	6	3	5	3	5	4	5	5	5	4	5	3	3	5	3	4	4	4	6	3	5	5	5	5	4	4	5	5	4	4	5	4	5	6	4	5	5	3	3	3	4	4	5	4	5	4	5	3	5	4	4	5	3	6	3	3	4	4	4	4	4	3	5	3	3	3	4	4	3
GGCAAT	6	5	5	3	4	5	5	4	6	6	5	5	4	4	5	5	5	6	4	4	5	4	4	5	4	5	3	6	5	5	5	3	5	5	4	3	5	5	5	4	4	5	5	4	3	5	5	3	3	4	3	5	5	6	5	4	4	5	5	4	4	6	3	5	3	4	4	3	3	4	3	4	3	4	5	4	3	3	4	3
GGCGTA	6	6	4	5	4	3	4	5	4	6	6	5	4	3	4	5	5	5	5	5	5	4	3	4	5	3	4	5	5	5	5	4	5	5	4	5	4	5	4	5	6	4	4	5	4	3	5	4	4	3	3	4	5	5	4	5	5	5	5	4	3	4	5	3	4	3	3	4	4	4	3	3	5	5	4	3	4	4	4	3	3
