# Supplementary material for: Not All Sequence Tags Are Created Equal: Designing and Validating Sequence Identification Tags Robust to Indels
Source: PLoS One. 2012 Aug 10;7(8):e42543. doi: 10.1371/journal.pone.0042543 (PMC3416851; doi:10.1371/journal.pone.0042543)
Supplement: Figure S9 — Pairwise Hamming distance comparisons between the 48 sequence tags provided as part of the Illumina TruSeq library preparation kits. The tags used within the DNA and RNA kits are a subset of those used within the smallRNA kit. The minimum expected Hamming distance of the set is three. The minimum observed Hamming distance of the set is two. (PDF) [file pone.0042543.s009.pdf]

Figure S9. Pairwise Hamming distance comparisons between the 48 sequence tags provided as part of the Illumina TruSeq library preparation kits. The tags used within the DNA and RNA kits are a subset of those used within the smallRNA kit. The minimum expected Hamming distance of the set is three. The minimum observed Hamming distance of the set is two.

		IDX1 ATCACG	IDX2 CGATGT	IDX3 TTAGGC	IDX4 TGACCA	IDX5 ACAGTG	IDX6 GCCAAT	IDX7 CAGATC	IDX8 ACTTGA	IDX9 GATCAG	IDX10 TAGCTT	IDX11 GGCTAC	IDX12 CTTGTA	IDX13 AGTCAA	IDX14 AGTTCC	IDX15 ATGTCA	IDX16 CCGTCC	IDX17 GTAGAG	IDX18 GTCCGC	IDX19 GTGAAA	IDX20 GTGGCC	IDX21 GTTTCG	IDX22 CGTACG	IDX23 GAGTGG	IDX24 GGTAGC	IDX25 ACTGAT	IDX26 ATGAGC	IDX27 ATTCCT	IDX28 CAAAAG	IDX29 CAACTA	IDX30 CACCGG	IDX31 CACGAT	IDX32 CACTCA	IDX33 CAGGCG	IDX34 CATGGC	IDX35 CATTTT	IDX36 CCAACA	IDX37 CGGAAT	IDX38 CTAGCT	IDX39 CTATAC	IDX40 CTCAGA	IDX41 GACGAC	IDX42 TAATCG	IDX43 TACAGC	IDX44 TATAAT	IDX45 TCATTC	IDX46 TCCCGA	IDX47 TCGAAG
IDX2	CGATGT	6																																														
IDX3	TTAGGC	5	4																																													
IDX4	TGACCA	5	4	4																																											
IDX5	ACAGTG	4	5	4	5																																										
IDX6	GCCAAT	4	5	6	6	5																																								
IDX7	CAGATC	5	5	5	6	5	5																																							
IDX8	ACTTGA	5	4	5	5	4	5	6																																						
IDX9	GATCAG	5	6	6	5	5	4	5	5																																					
IDX10	TAGCTT	6	5	5	4	5	5	3	6	4																																				
IDX11	GGCTAC	5	4	5	5	6	3	5	5	4	6																																			
IDX12	CTTGTA	5	5	4	5	4	6	4	4	5	5	6																																		
IDX13	AGTCAA	5	5	6	3	5	5	6	3	3	5	4	4																																	
IDX14	AGTTCC	4	4	5	4	5	6	5	3	5	6	3	5	3																																
IDX15	ATGTCA	3	5	5	4	5	6	5	3	6	5	5	4	4	3																															
IDX16	CCGTCC	5	4	5	5	5	5	3	4	6	5	4	5	6	3	3																														
IDX17	GTAGAG	4	5	3	5	3	4	6	6	3	6	4	4	5	6	5	6																													
IDX18	GTCCGC	4	5	3	5	6	4	5	5	4	5	3	5	5	5	5	5	4																												
IDX19	GTGAAA	4	6	5	5	6	3	4	5	4	5	4	4	4	6	3	5	3	4																											
IDX20	GTGGCC	4	6	3	5	5	5	4	6	5	5	4	4	6	4	3	3	3	3	3																										
IDX21	GTTTCG	3	5	5	5	5	5	6	4	3	6	4	4	5	3	3	4	3	4	4	3																									
IDX22	CGTACG	3	4	6	4	5	5	4	5	4	6	5	4	4	3	5	4	5	6	5	5	3																								
IDX23	GAGTGG	5	4	5	6	5	5	4	4	3	4	4	6	6	5	4	4	4	4	4	4	3	5																							
IDX24	GGTAGC	5	4	4	5	6	4	4	4	4	6	3	5	4	3	6	5	5	3	4	4	4	3	4																						
IDX25	ACTGAT	5	5	5	6	3	3	6	3	4	5	5	4	3	4	5	5	4	6	5	5	5	5	6	5																					
IDX26	ATGAGC	3	5	3	6	5	5	3	4	6	5	5	5	5	4	3	4	5	3	3	3	5	5	4	3	5																				
IDX27	ATTCCT	3	5	5	4	5	5	6	4	4	4	6	4	3	3	3	5	5	4	5	4	3	4	6	5	3	4																			
IDX28	CAAAAG	4	4	5	5	4	4	3	6	3	5	5	5	5	6	6	5	3	6	4	6	5	3	4	5	5	5	6																		
IDX29	CAACTA	6	4	5	3	4	6	3	5	4	3	6	3	4	6	5	5	5	5	5	6	6	5	5	6	6	6	5	3																	
IDX30	CACCGG	4	4	5	5	5	5	4	5	3	4	5	5	5	6	6	5	5	3	6	6	5	4	3	5	6	5	5	3	3																
IDX31	CACGAT	5	4	5	6	5	3	4	6	4	4	4	4	5	6	6	5	4	5	5	5	6	5	5	6	3	6	5	3	4	3															
IDX32	CACTCA	4	4	6	4	6	5	4	4	5	5	4	4	5	4	3	3	6	5	5	5	4	4	4	6	6	6	5	4	3	3	3														
IDX33	CAGGCG	4	5	5	5	4	6	3	6	4	4	6	4	6	5	4	3	4	6	5	3	4	3	3	6	5	5	5	3	4	3	3	3													
IDX34	CATGGC	6	4	3	6	5	6	3	4	4	5	5	3	5	4	6	4	5	4	6	4	5	4	4	3	4	4	5	4	4	3	3	4	3												
IDX35	CATTTT	6	3	6	6	5	5	3	4	4	3	5	3	5	4	5	4	6	6	6	6	4	4	4	5	4	6	4	4	3	4	3	3	4	3											
IDX36	CCAACA	4	4	5	3	4	4	4	4	6	6	6	4	5	5	4	3	5	6	4	5	5	3	6	5	5	5	5	3	3	5	5	3	4	5	5										
IDX37	CGGAAT	5	3	6	5	6	3	3	6	5	4	4	5	4	5	5	4	5	6	3	5	6	3	5	4	4	4	5	3	5	5	3	5	4	5	4	4									
IDX38	CTAGCT	4	3	3	4	4	5	5	6	6	5	6	3	6	5	4	4	3	5	5	3	4	4	6	6	4	5	3	4	4	5	3	4	3	4	4	3	4								
IDX39	CTATAC	5	3	3	5	5	5	4	5	5	6	3	4	5	4	4	3	3	4	4	4	4	5	5	5	5	4	5	3	4	5	4	4	5	4	4	4	4	3							
IDX40	CTCAGA	3	4	4	5	6	4	4	4	6	6	5	3	5	6	4	5	5	3	3	5	5	4	5	4	6	3	5	4	4	3	4	3	5	4	5	3	4	4	4						
IDX41	GACGAC	5	6	4	6	5	3	4	6	3	5	2	5	5	5	6	5	3	3	4	3	5	6	4	4	4	5	6	4	5	4	2	4	4	3	5	6	5	5	4	5					
IDX42	TAATCG	4	4	4	3	4	6	5	5	4	4	5	6	6	4	4	4	4	6	6	5	3	4	3	6	6	6	5	3	4	4	5	3	3	5	4	4	6	4	4	6	5				
IDX43	TACAGC	4	5	3	5	6	4	3	5	5	4	4	6	6	5	6	5	6	3	5	5	6	5	4	3	6	3	6	4	5	3	4	4	5	3	5	5	5	6	5	3	3	4			
IDX44	TATAAT	5	5	5	5	6	3	4	5	3	3	5	5	4	5	6	6	5	6	4	6	5	4	5	4	3	5	4	3	5	5	3	5	5	4	3	5	3	5	5	5	4	4	3		
IDX45	TCATTC	6	4	3	4	3	5	4	4	6	4	4	5	6	4	5	3	5	5	6	5	5	6	5	5	5	5	6	5	4	6	6	5	6	5	4	4	6	5	3	6	5	3	4	5	
IDX46	TCCCGA	5	5	4	3	5	4	6	3	5	4	5	5	4	6	5	5	6	3	5	6	6	6	5	5	5	5	5	6	4	3	5	4	6	5	6	4	6	6	6	3	5	5	3	5	4	
IDX47	TCGAAG	4	6	5	5	4	3	4	5	4	4	5	6	5	6	5	4	4	6	3	5	5	4	4	5	4	4	6	3	6	5	5	6	4	6	6	4	3	6	5	5	5	4	4	3	4	4	
IDX48	TCGGCA	5	6	4	3	4	5	5	4	6	4	6	4	5	5	3	3	5	6	4	3	5	5	5	6	4	5	5	6	5	6	5	4	3	5	6	3	5	4	6	5	5	4	5	5	4	3	3
