# Supplementary material for: Not All Sequence Tags Are Created Equal: Designing and Validating Sequence Identification Tags Robust to Indels
Source: PLoS One. 2012 Aug 10;7(8):e42543. doi: 10.1371/journal.pone.0042543 (PMC3416851; doi:10.1371/journal.pone.0042543)

Figure S10. Agarose gel image of *rbcLa* amplicons generated using fusion-style primers integrating 10 nucleotide, edit distance five sequence tags.

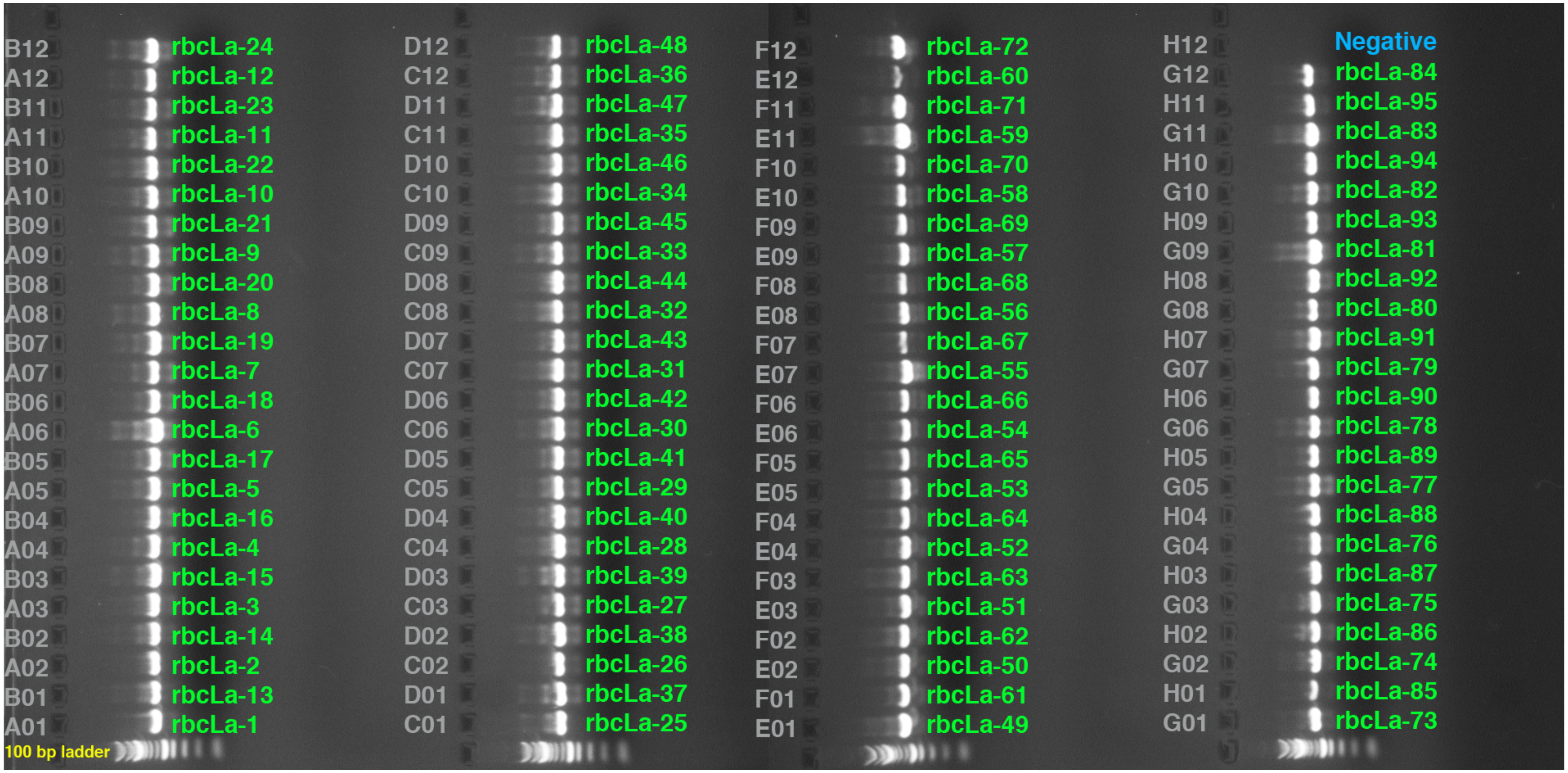

Supplement: Figure S10 — Agarose gel image of rbcLa amplicons generated using fusion-style primers integrating 10 nucleotide, edit distance five sequence tags. (PDF) [file pone.0042543.s010.pdf]
