# Supplementary material for: Not All Sequence Tags Are Created Equal: Designing and Validating Sequence Identification Tags Robust to Indels
Source: PLoS One. 2012 Aug 10;7(8):e42543. doi: 10.1371/journal.pone.0042543 (PMC3416851; doi:10.1371/journal.pone.0042543)
Supplement: Figure S11 — Fold difference in the average number of reads per well (across two plates) for PCR amplicons incorporating sequence tags designed using EDITTAG relative to the average number of reads per plate. (PDF) [file pone.0042543.s011.pdf]

Figure S11. Fold difference in the average number of reads per well (across two plates) for PCR amplicons incorporating sequence tags designed using EDITTAG relative to the average number of reads per plate.

	A	B	C	D	E	F	G	H
1	1.8	1.4	1.6	1.8	0.7	1.1	0.6	0.5
2	2.1	1.1	0.9	1.8	0.7	1.2	1.1	0.5
3	1.4	0.7	0.2	0.8	1.2	1.3	0.9	1.5
4	0.7	1.4	0.4	1.4	0.8	1.0	0.7	1.5
5	1.6	0.9	0.8	3.3	1.0	0.8	2.3	0.6
6	0.2	1.3	1.0	2.3	1.3	0.4	0.8	0.5
7	0.9	1.5	1.0	1.8	0.3	1.3	1.1	0.6
8	0.5	1.2	2.7	0.7	0.6	0.6	1.0	0.4
9	0.5	0.4	0.8	1.5	0.6	0.5	1.3	0.4
10	0.9	0.6	1.1	1.1	0.8	0.4	1.4	0.7
11	1.2	0.9	1.1	0.7	1.0	0.6	0.7	1.2
12	0.7	0.7	1.3	0.3	0.5	0.4	0.2	NEG

Average reads per well	20.9
Mean fold difference	1.0
Mean fold diff. 95 CI	0.1
