# Supplementary material for: Not All Sequence Tags Are Created Equal: Designing and Validating Sequence Identification Tags Robust to Indels
Source: PLoS One. 2012 Aug 10;7(8):e42543. doi: 10.1371/journal.pone.0042543 (PMC3416851; doi:10.1371/journal.pone.0042543)

Figure S12. Comparison of the number of reads returned for libraries incorporating adapters having six nucleotide Epicentre Nextera indices or EDITTAG-designed indices.

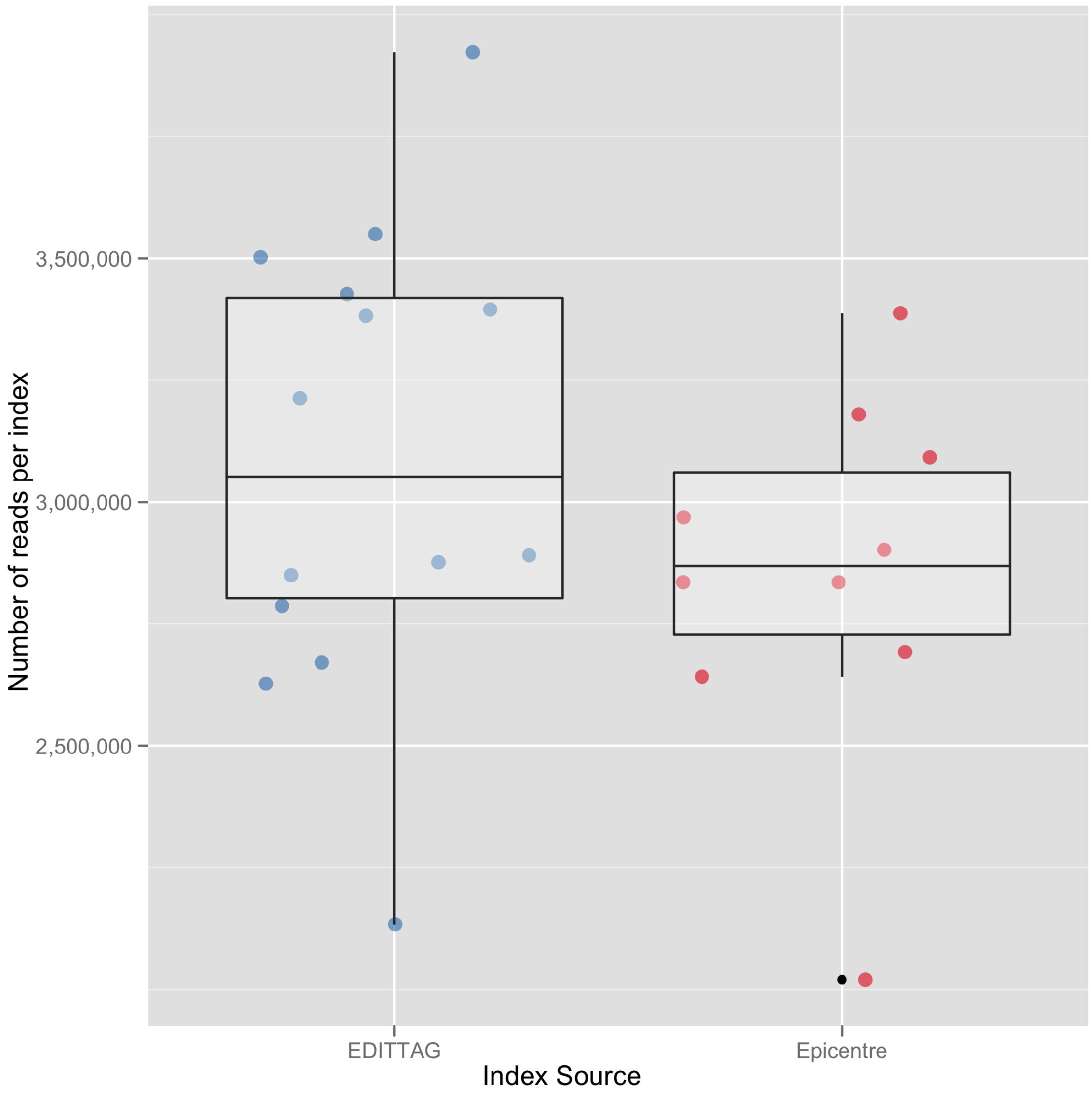

Supplement: Figure S12 — Comparison of the number of reads returned for libraries incorporating adapters having six nucleotide Epicentre Nextera indices or EDITTAG-designed indices. (PDF) [file pone.0042543.s012.pdf]
