# Supplementary material for: Not All Sequence Tags Are Created Equal: Designing and Validating Sequence Identification Tags Robust to Indels
Source: PLoS One. 2012 Aug 10;7(8):e42543. doi: 10.1371/journal.pone.0042543 (PMC3416851; doi:10.1371/journal.pone.0042543)

Figure S13. Comparison of the number of reads returned for libraries prepared by (A) ligating an adapter pool to DNA fragments and (B) ligating individual adapters to DNA fragments. Although the number of reads are different between runs, note that variance is much higher (>2 orders of magnitude) in (A). Additionally, some adapters (e.g., BFIDT-012) performing poorly in (A) function well in (B).

A)

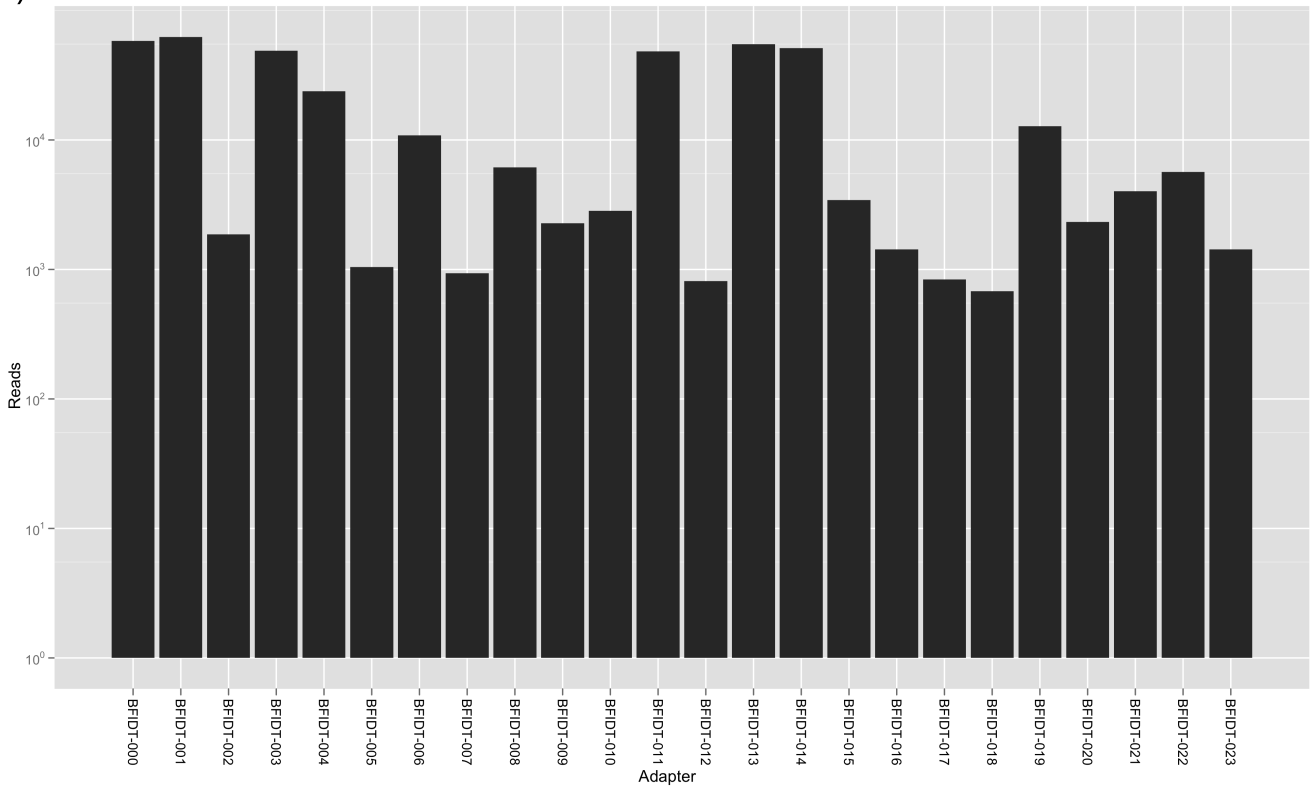

B)

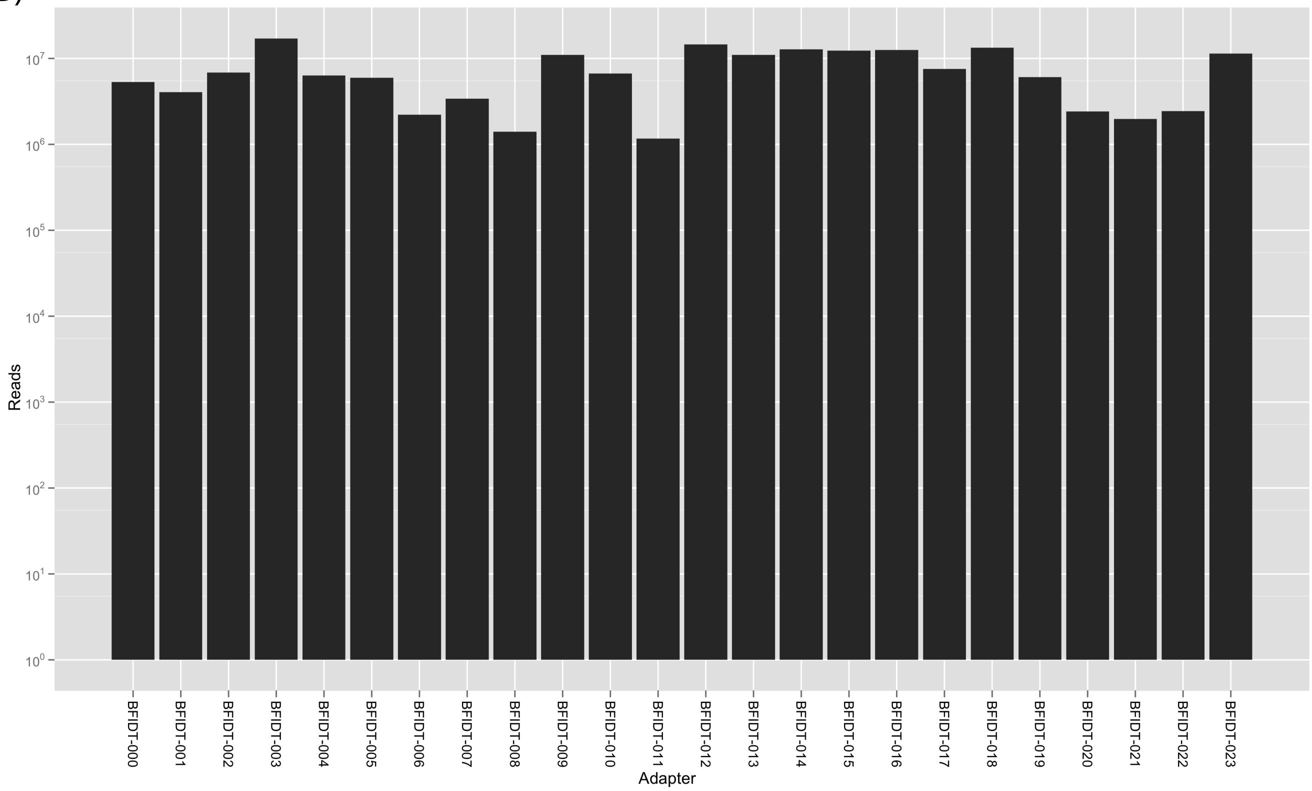

Supplement: Figure S13 — Comparison of the number of reads returned for libraries prepared using two methods. We prepared the first library (A) by ligating an adapter pool to DNA fragments, and we prepared the second (B) by ligating individual adapters to DNA fragments. Although the numbers of reads are different between runs, note that variance is much higher (>2 orders of magnitude) in (A). Additionally, some adapters (e.g., BFIDT-012) performing poorly in (A) function well in (B). (PDF) [file pone.0042543.s013.pdf]
