# Supplementary material for: Not All Sequence Tags Are Created Equal: Designing and Validating Sequence Identification Tags Robust to Indels
Source: PLoS One. 2012 Aug 10;7(8):e42543. doi: 10.1371/journal.pone.0042543 (PMC3416851; doi:10.1371/journal.pone.0042543)

Figure S14. Comparison of the number of reads returned for 64 libraries incorporating adapters having 10 nucleotide EDITTAG-designed indices versus 12 libraries incorporating Illumina TruSeq adapters. Outliers represent failed enrichments.

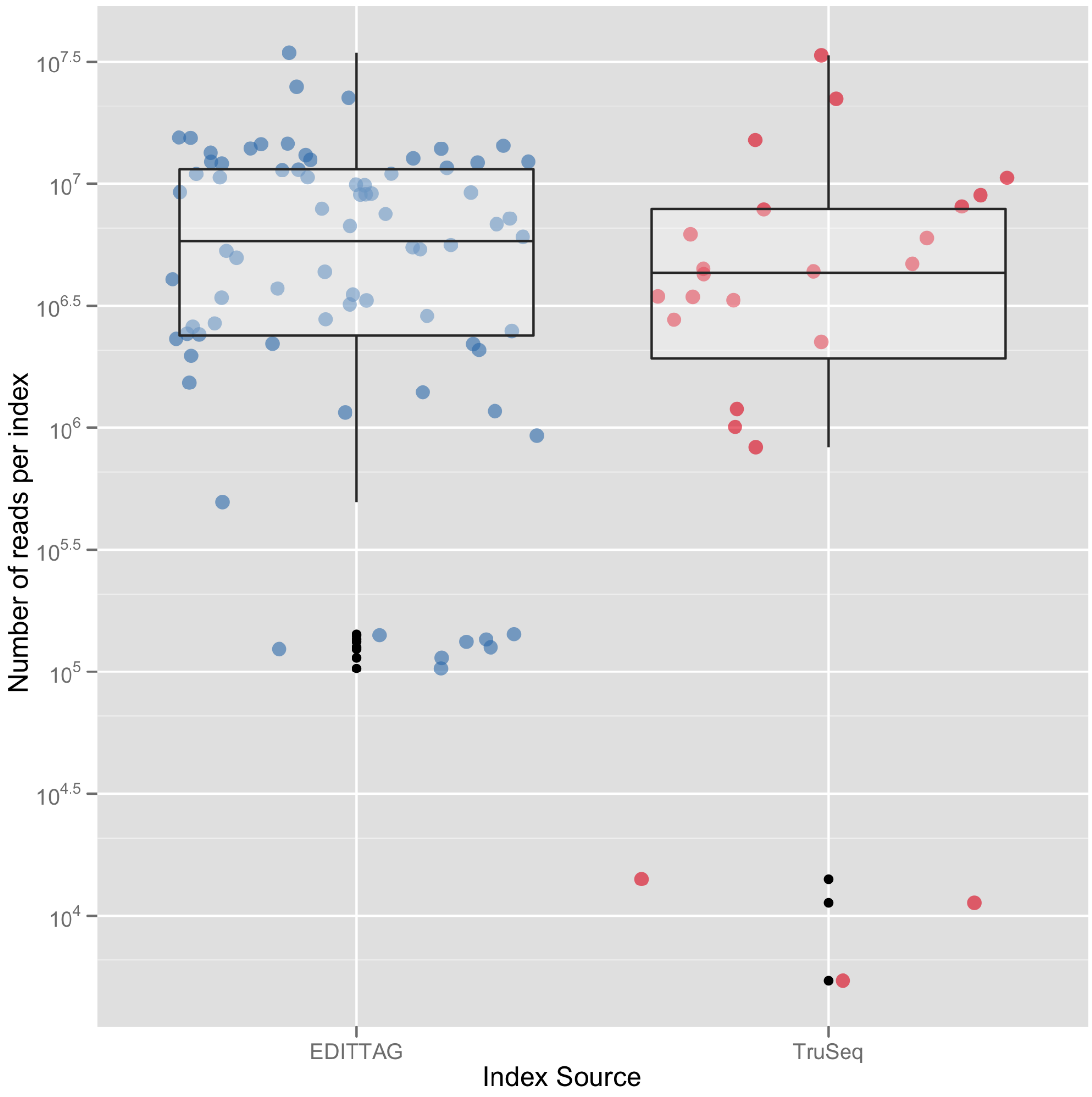

Supplement: Figure S14 — Comparison of the number of reads returned for 64 libraries incorporating adapters having 10 nucleotide EDITTAG-designed indices versus 12 libraries incorporating Illumina TruSeq adapters. Outliers represent failed enrichments. (PDF) [file pone.0042543.s014.pdf]
