# Supplementary material for: Not All Sequence Tags Are Created Equal: Designing and Validating Sequence Identification Tags Robust to Indels
Source: PLoS One. 2012 Aug 10;7(8):e42543. doi: 10.1371/journal.pone.0042543 (PMC3416851; doi:10.1371/journal.pone.0042543)
Supplement: Table S1 — The counts of sequence tags within commercial and non-commercial sets having a minimum edit distance of three or five. (PDF) [file pone.0042543.s015.pdf]

Table S1. The counts of sequence tags within commercial and non-commercial sets having a minimum edit distance of three or five.

Set Name	Length (nt)	N_{tags}	Count of tags ≥ edit distance 3	Count of tags ≥ edit distance 5
Illumina TruSeq sRNA	6	48	36	3
Hamady et al. 2007[5]	8	1544	164	19
Meyer et al. 2010[1]	6	75	49	4
Meyer et al. 2010[1]	8	711	429	20
Adey et al. 2010[6]	9	96	92	15
Illumina TruSeq RNA and DNA	6	27	21	3
Meyer et al. 2008[3]	7	52	24	3
Meyer et al. 2008	8	130	65	6
Qiu et al. 2003	6	21	21	3
Frank 2009[2]	6	81	72	4
Illumina Nextera DNA[4]	8	8/12	8/12	4/5
Frank 2009[2]	8	760	557	18
Roche 454 MID Extended	10	151	151	17
Roche 454 RL-MID Extended	10	132	132	13
