# Supplementary material for: Not All Sequence Tags Are Created Equal: Designing and Validating Sequence Identification Tags Robust to Indels
Source: PLoS One. 2012 Aug 10;7(8):e42543. doi: 10.1371/journal.pone.0042543 (PMC3416851; doi:10.1371/journal.pone.0042543)
Supplement: Figure S3 — Pairwise edit distances between 96 sequence tags described in Supplementary Table 4 of Adey et al. [41]. The minimum expected edit distance of the set is four. The minimum observed edit distance of the set is two. (PDF) [file pone.0042543.s003.pdf]

Figure S3. Pairwise edit distances between 96 sequence tags described in Adey *et al.* 2010, Supplementary Table 4. The minimum expected edit distance of the set is four. The minimum observed edit distance of the set is two.

	TACGAAGTC	GACGAGATT	ACCGTAAGA	TAGTGGCAA	CATTAACGC	TCGTTGAAG	TAGTACGCT	CTCAGATCA	TTCACCGTA	GTCATGCAT	AGGACAGTT	ATGGTGTCT	GGATGTTCT	CTTATCCAG	GTAAGTCAC	TTCAGTGAG	CTCGTAATG	CATGTCTCA	AATCGTGGA	GTATCAGTC	AGCAGATGT	TCCTAACGT	AACAGTCCA	CCTTGAGAA	TTAAGCCTG	TTAGACCAC	TGTCTAGTG	TAGATCGAG	TGAATGCCA	GTGCAATGT	AGTGGCATA	ATGATCGGT	AGTCTACCT	GATCAACTG	ATCGGTAGT	CGTATGATG	TTACTGACG	CTGTCGTAA	TCAACTGGT	ATCGATCTC	GCAACTATG	GATGACTTC	GACGTTACA	CATCTGCTA	ATTAGTCGG	TAGCGTACT	CCAAGCAAT	CCGTAATTG	AGAATTGCC	ACCTGTAAC	CATCAGTGT	GAATCCTCA	GCTGTATAC	GAAGGCTAT	GGAATCGAT	GCTTATGGT	TGACGCATT	CACGATTCT	TATTGCCTC	AAGTCAGAG	ATAGCTGAG	TGCTCACAA	GTCTTCTGA	TTGCCGATT	CTCGAATAC	TGGCTTCTA	AAGGCCATT	AAGTTGACC	CTGAACTGA	CTAGGTGTA	CCATCTTAG	CTACGACAT	TCCAACATG	GCTATCATC	ACAGCTTCA	AGTCATTGC	AGATCTCGA	ATGCTCTTG	TTAGTGCGT	TCCTAGTTC	GGTGCATTA	ACTGAGGAT	TAGCAGTCA	TCACTCGAA	ACCAATCAG	GATATGGAC	TGAGAGATC	TGCCATTAG	ACTAACGCA	ATGTAGCAC	GGTCGATAT	GCGAGTTAT	GACTGAGTA	AGATACTCC	GCTAGAGTT
GACGAGATT	4
ACCGTAAGA	5	6
TAGTGGCAA	6	7	6
CATTAACGC	5	7	5	6
TCGTTGAAG	6	6	5	4	5
TAGTACGCT	5	6	6	4	4	6
CTCAGATCA	6	5	6	6	6	6	6
TTCACCGTA	5	7	6	7	6	6	6	5
GTCATGCAT	6	5	7	5	7	5	5	5	5
AGGACAGTT	4	4	6	6	7	7	4	7	5	6
ATGGTGTCT	5	6	6	5	7	6	5	6	7	5	5
GGATGTTCT	6	5	8	7	7	7	6	6	8	5	5	4
CTTATCCAG	7	8	7	6	5	5	7	5	6	4	7	7	7
GTAAGTCAC	6	6	6	5	6	7	5	4	6	4	6	6	5	5
TTCAGTGAG	6	6	5	5	6	4	6	4	4	4	7	6	7	5	4
CTCGTAATG	5	5	4	7	5	4	6	5	6	5	7	6	7	5	6	4
CATGTCTCA	6	7	6	5	5	7	5	5	7	6	7	4	4	4	5	7	6
AATCGTGGA	6	6	4	5	6	5	7	6	6	5	7	5	6	7	7	5	6	5
GTATCAGTC	4	5	7	7	6	7	6	5	5	4	4	6	5	5	4	5	6	6	6
AGCAGATGT	5	4	5	7	6	6	6	4	6	6	4	5	5	8	7	5	6	7	6	6
TCCTAACGT	4	6	4	6	4	5	4	6	4	5	7	6	7	6	7	6	4	7	7	6	5
AACAGTCCA	5	6	5	5	7	7	6	4	6	5	5	6	6	5	5	5	7	4	4	5	5	7
CCTTGAGAA	7	6	4	5	5	4	7	5	6	6	7	7	7	6	7	6	6	6	5	6	7	6	7
TTAAGCCTG	6	7	8	6	6	7	5	6	4	5	7	6	6	4	4	4	6	6	6	6	6	5	6	7
TTAGACCAC	5	7	7	5	5	6	5	4	5	6	7	7	7	4	4	5	7	6	7	6	7	6	5	6	4
TGTCTAGTG	5	5	7	7	6	5	6	6	5	6	5	6	6	6	6	4	4	6	6	4	5	5	7	6	5	7
TAGATCGAG	5	6	7	4	6	4	4	4	5	6	6	7	7	4	5	4	6	6	5	6	6	6	5	6	5	4	5
TGAATGCCA	5	7	7	4	6	6	5	5	6	5	7	5	5	5	5	6	6	5	6	6	6	7	5	7	4	5	6	5
GTGCAATGT	5	5	6	6	6	6	6	5	5	4	5	5	6	7	6	5	4	7	7	5	3	4	7	8	6	7	5	7	5
AGTGGCATA	7	6	5	2	7	6	5	6	6	5	4	5	6	7	6	7	6	5	5	5	5	7	6	6	6	7	6	6	6	5
ATGATCGGT	6	6	6	7	5	7	5	6	5	5	4	4	5	5	6	6	7	5	5	4	5	6	6	7	6	6	5	4	6	5	6
AGTCTACCT	7	6	6	6	6	7	4	6	5	4	5	5	5	6	6	8	6	5	6	6	5	5	6	7	6	6	4	6	5	5	5	6
GATCAACTG	5	5	7	7	4	6	6	6	5	5	6	7	5	6	6	6	5	6	6	4	5	6	6	7	5	7	4	5	7	4	7	5	5
ATCGGTAGT	5	5	4	6	7	5	5	6	6	5	5	4	6	7	6	4	4	7	4	6	4	5	5	7	7	7	5	6	8	5	5	4	5	6
CGTATGATG	6	5	5	7	5	3	5	5	7	4	6	7	5	4	6	5	4	6	7	4	4	6	8	5	5	7	4	5	5	5	5	6	6	5	6
TTACTGACG	5	6	6	5	5	4	5	5	5	6	7	5	6	5	5	3	5	7	7	5	6	4	6	7	4	5	5	5	4	6	7	6	6	6	6	4
CTGTCGTAA	7	7	6	4	7	5	6	5	5	6	6	5	7	5	6	5	4	3	5	5	7	6	6	5	7	6	5	5	6	6	5	5	6	8	6	6	7
TCAACTGGT	6	7	7	6	6	6	5	6	5	5	5	7	6	6	6	5	6	7	5	6	5	4	7	7	5	6	5	6	5	4	7	4	6	4	5	6	5	7
ATCGATCTC	4	5	6	7	5	6	6	4	5	5	6	4	5	6	5	6	5	4	5	5	4	6	5	7	6	4	6	5	6	5	6	4	4	4	4	6	6	6	6
GCAACTATG	7	5	6	8	6	6	7	7	6	5	6	8	6	6	5	6	5	6	7	5	5	6	7	8	5	6	5	6	7	5	6	7	6	4	7	5	5	7	4	6
GATGACTTC	4	4	8	6	5	7	6	6	6	5	5	5	4	7	5	7	7	4	7	4	6	6	6	7	6	5	6	6	6	5	5	5	6	4	7	5	6	6	7	4	5
GACGTTACA	6	4	4	5	6	5	6	5	7	5	6	6	4	6	4	6	5	4	5	6	7	6	4	6	8	7	7	6	5	7	6	7	5	6	5	6	5	5	8	6	6	5
CATCTGCTA	7	6	6	5	5	6	5	4	5	5	7	5	5	5	7	6	5	3	4	5	6	7	5	5	6	7	5	6	5	7	5	6	5	4	5	4	6	5	4	5	5	6	6
ATTAGTCGG	6	7	6	6	4	6	5	5	6	6	7	5	7	4	4	4	6	6	4	5	5	6	4	7	4	5	5	4	6	6	6	3	6	5	4	6	5	6	6	5	6	6	7	6
TAGCGTACT	4	5	5	5	7	6	4	6	5	6	6	4	5	7	5	6	5	6	5	5	5	5	5	7	6	5	5	5	6	6	6	6	4	5	4	7	5	5	5	5	6	6	4	5	7
CCAAGCAAT	6	5	6	6	7	6	7	5	7	4	6	8	6	5	5	6	5	6	8	6	6	5	6	5	5	6	8	6	5	6	6	6	7	6	6	6	7	6	5	7	4	7	7	6	7	6
CCGTAATTG	5	5	4	7	5	5	6	6	8	7	6	7	7	6	6	6	2	6	7	6	6	5	8	5	6	8	5	7	7	5	7	7	7	5	6	4	7	5	6	6	5	6	6	7	6	6	5
AGAATTGCC	5	6	7	6	6	7	5	7	7	6	5	6	5	7	5	6	7	7	6	5	5	8	5	8	6	7	6	5	3	6	6	5	5	6	7	6	6	8	5	6	6	5	5	6	6	6	7	5
ACCTGTAAC	6	6	3	6	5	5	7	6	6	6	7	6	6	6	5	5	5	5	6	6	6	5	5	5	8	7	7	7	7	7	6	7	5	7	4	6	6	5	7	5	6	7	5	5	6	5	5	5	6
CATCAGTGT	6	5	6	7	4	7	6	4	5	5	5	5	6	6	6	4	5	5	4	4	3	5	5	6	6	8	4	6	7	4	7	5	6	4	3	5	7	5	4	5	5	5	7	4	4	5	6	5	6	6
GAATCCTCA	7	7	7	6	6	8	5	6	6	5	7	7	4	6	5	7	7	4	4	4	7	7	5	7	5	6	7	6	3	7	6	7	6	5	7	6	7	5	7	6	6	5	5	5	6	6	7	7	5	7	6
GCTGTATAC	6	6	5	7	6	6	6	6	7	5	7	6	5	5	5	6	5	5	6	5	5	6	6	5	8	6	6	7	6	5	5	7	6	6	6	5	6	4	8	6	5	4	4	6	7	6	6	5	7	3	7	7
GAAGGCTAT	7	4	7	5	8	8	6	7	7	4	6	5	4	7	4	7	7	5	7	6	5	8	5	7	5	5	6	6	4	6	5	6	5	6	5	6	7	6	6	6	4	4	5	5	7	5	4	8	6	7	6	4	5
GGAATCGAT	7	5	7	6	7	7	5	6	6	4	5	6	5	5	4	6	7	7	6	5	5	6	6	7	6	6	6	4	4	4	6	4	6	5	6	4	6	6	5	6	4	6	7	6	6	6	4	6	4	8	6	5	6	4
GCTTATGGT	7	6	6	7	5	6	5	6	6	4	6	6	5	5	6	6	6	7	5	5	4	4	8	5	6	8	6	7	7	5	8	4	6	6	6	4	6	6	4	7	5	6	6	5	5	6	7	5	6	6	5	7	4	7	5
TGACGCATT	4	3	7	5	7	6	4	6	5	4	5	6	5	6	6	7	6	6	7	6	5	6	7	7	4	5	5	5	5	5	4	5	6	6	6	5	5	5	6	6	6	5	5	6	7	4	4	6	7	7	6	6	6	5	5	6
CACGATTCT	4	4	7	7	6	7	5	4	7	5	6	4	4	6	7	7	5	4	6	6	5	6	5	7	7	7	7	6	7	6	7	5	6	6	5	6	6	7	6	4	6	4	4	5	6	4	6	5	5	7	4	6	6	5	6	6	5
TATTGCCTC	5	7	8	4	4	6	4	7	5	5	7	5	6	6	5	7	7	4	6	5	8	6	6	6	4	4	6	6	4	7	5	6	5	5	7	6	5	7	6	4	8	4	7	4	5	5	7	7	5	6	6	4	6	6	7	7	5	6
AAGTCAGAG	6	6	4	5	6	5	5	6	7	5	4	7	8	6	5	5	6	5	5	5	4	7	5	5	6	6	4	3	7	6	5	5	5	5	5	5	6	5	7	7	6	7	5	6	4	6	5	5	6	6	4	6	6	5	5	7	7	8	7
ATAGCTGAG	6	7	5	5	6	4	6	5	6	6	6	5	7	5	5	3	5	5	5	5	5	6	6	6	5	5	5	3	7	6	5	5	7	5	4	5	3	6	5	5	5	6	7	5	4	5	6	7	5	6	5	6	7	5	6	7	7	7	6	3
TGCTCACAA	6	7	6	4	6	5	6	5	5	5	6	6	7	5	6	6	6	5	7	6	6	4	6	5	7	6	5	5	5	6	5	6	5	5	7	7	5	4	7	6	6	6	5	6	8	5	6	7	7	6	7	6	5	7	6	6	5	8	6	5	6
GTCTTCTGA	8	7	5	7	7	5	7	5	5	5	8	6	5	6	6	5	5	5	5	5	6	6	6	5	6	7	4	6	6	5	6	5	5	4	6	5	5	5	5	5	6	6	5	4	6	7	8	6	7	6	6	4	5	6	5	4	7	6	6	6	5	6
TTGCCGATT	6	5	7	6	7	5	5	5	4	6	5	5	6	6	7	6	6	7	7	6	4	6	8	6	5	5	5	5	6	4	5	5	6	7	5	6	4	5	6	5	6	6	7	6	6	4	7	6	8	7	6	8	6	6	6	6	3	6	5	6	5	6	6
CTCGAATAC	4	5	5	7	5	6	6	4	6	6	7	6	7	6	5	5	3	6	6	6	6	6	6	5	7	4	6	6	5	5	5	7	7	6	5	6	6	5	6	4	6	5	5	5	7	5	6	4	6	5	5	7	3	6	6	7	6	4	6	7	6	6	6	6
TGGCTTCTA	7	7	7	5	8	5	6	6	5	6	6	4	5	7	7	7	6	5	6	7	7	6	6	7	6	7	4	5	4	6	5	7	5	6	6	6	6	5	6	5	6	5	5	4	7	4	8	7	6	7	7	5	5	6	5	6	5	5	6	7	6	4	3	5	7
AAGGCCATT	6	4	6	5	8	7	5	7	6	6	3	5	7	7	6	8	6	6	7	7	5	8	5	8	5	5	6	6	5	5	4	5	5	6	5	7	8	7	7	6	6	5	6	7	7	5	5	6	7	7	6	6	7	3	6	7	4	6	5	5	6	7	8	4	7	6
AAGTTGACC	6	6	6	5	4	4	5	7	7	7	7	4	6	7	5	6	7	6	5	6	6	7	5	6	7	6	7	5	6	8	5	6	4	6	6	5	5	6	7	5	7	5	4	6	6	5	7	7	3	5	7	6	6	6	6	7	7	6	4	4	4	6	7	6	7	7	5
CTGAACTGA	6	7	5	7	4	6	6	4	5	6	6	6	7	5	5	5	4	4	6	6	6	5	7	6	5	6	6	5	4	4	6	4	7	4	7	5	5	4	4	5	6	5	7	5	6	7	6	4	5	6	5	5	5	6	5	6	6	6	7	6	4	6	4	6	4	6	7	7
CTAGGTGTA	5	6	7	5	7	5	6	4	5	6	6	4	6	6	5	5	5	4	5	5	5	6	6	5	5	6	6	5	6	6	5	6	7	7	4	5	6	4	6	5	6	7	6	4	6	5	6	6	6	6	4	7	5	5	7	5	6	6	6	7	4	7	6	6	5	5	6	6	4
CCATCTTAG	7	7	6	7	5	5	8	5	5	6	8	7	5	4	6	5	5	5	5	6	7	6	6	5	6	7	6	5	7	8	7	7	7	6	5	5	5	4	5	6	4	6	6	4	7	6	5	4	7	5	5	5	5	6	6	6	7	6	7	5	4	6	5	8	6	6	8	7	5	5
CTACGACAT	4	4	7	5	6	6	5	4	6	4	5	6	6	5	4	6	4	6	7	6	6	5	6	5	5	4	7	5	6	5	6	6	5	6	5	5	4	5	6	5	6	5	5	6	6	4	4	6	8	7	6	6	6	5	6	6	4	4	6	7	6	5	7	5	4	7	5	7	6	5	6
TCCAACATG	5	5	6	7	7	5	6	6	4	5	6	8	8	6	7	5	4	8	7	6	5	3	7	7	4	6	5	5	7	5	6	7	6	4	7	5	6	7	4	5	3	6	7	7	7	6	4	5	7	6	5	7	7	7	6	7	5	7	6	7	7	5	6	6	5	6	6	8	5	7	4	4
GCTATCATC	6	5	6	7	6	6	7	5	6	4	6	6	5	5	4	7	5	5	7	2	6	5	7	6	6	6	6	6	7	6	5	6	5	5	7	4	6	5	6	4	4	4	6	6	6	6	5	6	6	5	5	4	3	6	4	4	6	6	5	7	7	5	5	6	5	5	6	6	6	6	5	5	4
ACAGCTTCA	6	5	5	6	7	6	7	4	5	6	6	5	5	6	6	6	7	4	5	6	5	7	3	7	6	6	8	6	6	7	5	7	6	6	6	7	6	5	5	5	5	5	4	4	6	5	6	7	5	5	6	4	6	6	7	7	6	4	5	6	4	6	6	7	7	4	6	5	6	5	4	6	6	5
AGTCATTGC	6	6	6	7	5	6	5	6	6	4	5	6	6	7	5	5	6	6	4	5	4	7	5	8	7	7	4	6	6	4	5	5	4	5	5	5	6	6	6	4	6	5	5	6	5	6	7	4	3	6	4	6	6	7	6	5	6	6	6	4	6	6	4	6	5	6	5	6	6	7	6	7	7	6	6
AGATCTCGA	6	6	6	5	6	7	6	6	6	6	6	5	5	6	6	7	8	4	3	6	5	6	5	7	7	6	5	4	5	7	5	4	4	5	6	6	6	6	5	4	6	6	6	4	5	7	7	7	5	6	6	4	6	6	4	6	6	5	7	5	4	4	4	7	8	5	7	6	4	6	5	7	7	6	4	4
ATGCTCTTG	7	6	7	7	7	6	6	7	6	6	5	4	5	5	7	6	5	4	6	6	5	6	7	8	5	7	4	5	7	5	5	4	5	5	5	6	5	5	6	4	5	4	7	5	5	6	7	5	6	6	6	6	6	6	7	6	5	6	5	5	4	5	5	5	7	5	5	6	5	6	5	6	5	6	5	4	4
TTAGTGCGT	6	6	6	4	6	6	3	6	5	4	6	4	5	5	5	3	6	7	5	5	6	5	6	7	4	4	6	5	4	6	5	5	6	8	5	6	3	5	4	6	7	6	7	6	3	5	6	7	6	8	5	7	7	5	5	4	5	6	4	6	4	7	6	5	7	6	6	6	6	4	7	5	7	6	6	6	6	6
TCCTAGTTC	4	5	6	6	6	5	5	5	6	6	6	6	5	6	6	5	5	5	7	5	6	4	5	6	6	6	4	7	7	6	7	6	6	7	5	5	6	6	5	5	6	5	6	6	6	6	6	4	7	4	5	7	5	8	7	5	6	5	5	7	7	6	6	5	5	6	7	6	7	5	6	6	4	5	5	6	8	5	5
GGTGCATTA	6	5	6	5	7	7	6	6	6	5	5	6	5	7	6	6	6	5	6	5	6	8	7	6	7	7	5	7	6	4	3	7	6	5	7	6	7	5	8	6	6	4	6	6	8	6	6	5	7	7	6	5	4	6	6	6	4	6	6	6	6	5	5	5	6	4	5	7	5	6	6	7	7	5	5	4	6	5	6	6
ACTGAGGAT	5	4	4	5	6	6	5	5	7	5	5	5	7	6	6	5	6	7	4	7	4	5	6	4	6	6	7	6	7	6	4	4	6	7	5	5	5	5	6	6	6	6	6	6	6	6	5	7	7	5	5	7	5	6	6	4	5	5	7	5	5	6	6	5	5	8	6	6	5	5	7	5	6	5	6	6	6	6	6	6	6
TAGCAGTCA	3	6	6	4	7	6	4	4	5	6	5	5	6	7	4	4	6	5	5	3	4	7	3	6	6	5	5	4	4	5	5	6	6	6	6	7	5	5	6	6	7	6	5	5	5	3	6	6	5	5	4	5	6	6	6	8	5	5	5	5	5	5	7	5	6	4	6	5	5	4	7	5	6	5	4	5	5	6	6	5	5	7
TCACTCGAA	6	6	6	4	6	4	5	5	3	5	7	8	8	5	5	4	6	5	6	6	7	5	5	5	6	5	5	4	5	6	6	5	7	6	6	7	4	4	4	6	6	7	6	5	5	6	5	7	6	5	6	6	6	6	4	7	5	7	6	5	5	4	5	5	6	5	7	6	5	6	5	6	6	6	5	6	4	6	5	5	7	6	5
ACCAATCAG	5	6	4	7	6	6	7	4	7	5	6	7	8	4	5	4	6	6	7	6	5	5	4	6	6	6	7	5	6	6	7	5	6	4	5	5	6	7	6	4	5	7	6	7	4	7	4	5	6	4	6	6	6	7	5	6	7	5	8	5	5	6	6	7	5	7	7	7	6	7	4	6	3	5	5	6	6	6	7	6	7	5	4	5
GATATGGAC	6	6	7	5	5	6	6	7	7	4	7	6	5	5	4	6	8	6	4	4	6	7	6	5	7	6	6	4	5	7	6	5	7	6	6	5	5	6	6	6	6	5	5	5	6	6	7	7	4	5	6	5	4	5	4	4	7	7	5	6	5	7	6	7	6	7	7	5	6	6	6	6	8	4	7	5	4	7	6	6	6	5	7	6	7
TGAGAGATC	4	3	7	6	7	6	5	5	6	6	5	5	5	7	5	5	6	7	7	5	5	7	6	6	5	4	5	5	5	6	5	5	7	6	6	5	5	7	6	5	6	4	6	7	7	5	6	7	6	7	6	6	6	6	5	7	4	6	5	6	6	6	8	5	5	6	6	5	6	6	8	5	5	5	6	6	6	7	5	4	6	4	4	6	7	5
TGCCATTAG	6	7	5	7	6	5	7	6	5	5	6	7	6	6	6	4	6	6	7	6	6	5	7	6	6	6	4	5	6	5	5	6	7	6	6	5	5	5	5	6	4	6	5	7	7	6	7	5	6	6	6	7	5	7	6	6	4	5	7	5	5	4	6	4	5	4	5	8	6	7	4	6	4	5	7	4	6	4	7	5	4	7	5	6	4	6	6
ACTAACGCA	5	7	4	6	3	7	4	5	5	5	6	6	8	5	5	7	6	5	5	6	6	4	5	5	6	5	7	6	5	7	5	5	5	6	7	7	5	5	5	5	6	6	5	5	5	6	5	6	5	6	7	5	6	6	6	5	5	6	6	6	5	5	6	7	6	7	7	6	4	5	6	4	5	5	4	5	5	7	6	6	7	4	5	5	4	6	8	8
ATGTAGCAC	5	6	5	4	4	5	5	6	7	5	6	5	6	5	4	6	6	4	5	5	6	6	6	6	6	4	6	6	5	6	4	5	5	6	5	6	6	4	8	4	7	5	5	5	4	6	6	6	5	4	6	7	4	6	6	7	5	7	5	4	5	5	7	6	5	7	6	4	6	6	7	6	6	5	6	5	6	6	5	5	6	4	5	7	5	4	5	6	5
GGTCGATAT	6	4	7	6	7	5	6	5	7	4	5	5	4	7	6	6	5	7	6	6	4	7	8	5	8	6	5	6	7	4	4	6	4	5	4	5	7	4	6	4	5	5	5	5	7	5	6	6	8	7	5	6	4	4	4	5	4	5	7	5	6	5	6	4	4	6	5	6	7	6	6	4	6	5	8	5	6	7	7	7	4	5	7	6	6	6	5	5	7	6
GCGAGTTAT	6	3	6	6	7	6	7	6	7	5	6	5	3	7	4	5	5	6	7	6	4	7	6	6	7	7	6	6	7	5	6	6	6	7	5	5	7	6	5	6	4	5	5	7	6	5	4	5	6	5	6	7	4	4	4	5	6	4	8	7	6	7	6	6	5	6	6	6	6	5	5	5	6	4	5	6	7	7	6	5	5	4	5	6	5	6	5	4	7	6	4
GACTGAGTA	4	3	5	5	6	7	6	5	5	5	5	6	6	7	5	6	6	5	5	4	5	5	5	4	7	7	4	6	5	6	5	6	6	5	5	5	5	5	5	6	5	4	4	4	6	6	7	6	6	6	5	5	4	5	5	5	5	5	5	5	5	5	5	6	5	5	7	6	5	5	6	6	7	6	5	6	5	7	5	5	4	3	5	5	6	5	5	6	4	5	5	4
AGATACTCC	5	6	7	7	6	8	4	6	8	7	5	5	4	6	5	8	7	5	6	5	5	7	4	8	7	5	6	5	5	7	6	5	4	5	6	6	6	7	6	4	6	3	6	6	5	5	7	6	3	6	6	4	6	6	6	7	6	5	6	7	6	6	6	8	6	6	7	5	5	7	6	7	7	5	5	4	3	5	7	5	6	7	5	7	6	5	5	6	5	5	7	7	6
GCTAGAGTT	5	3	6	7	7	6	5	5	6	6	4	5	6	7	5	6	5	7	6	4	4	5	7	4	6	6	5	6	8	5	6	6	6	5	6	5	7	6	5	6	5	4	7	7	6	5	5	5	7	7	5	7	5	5	5	4	6	5	6	6	6	6	6	5	5	7	7	7	6	4	7	4	6	4	6	7	7	7	5	5	5	4	5	7	7	5	5	6	5	7	4	4	3	7
AATGTAGCG	5	6	5	5	4	6	4	7	7	7	6	5	5	6	6	6	5	4	4	6	6	6	5	6	6	7	4	5	6	6	6	5	4	5	5	6	5	6	7	6	6	6	5	5	4	5	7	6	5	4	5	6	5	6	7	6	7	6	6	3	5	7	7	7	7	8	6	4	6	6	6	7	7	6	6	5	5	5	5	7	6	5	5	7	6	5	7	7	5	3	7	8	5	6	6
